# Supplementary material for: COVID-19 outbreak–related psychological distress among healthcare trainees: a cross-sectional study in China
Source: BMJ Open. 2020 Oct 20;10(10):e041671. doi: 10.1136/bmjopen-2020-041671 (PMC7577057; doi:10.1136/bmjopen-2020-041671)
Supplement: Supplementary data [file bmjopen-2020-041671supp001.pdf]

Supplementary

Figure 1 Research Flow Chart

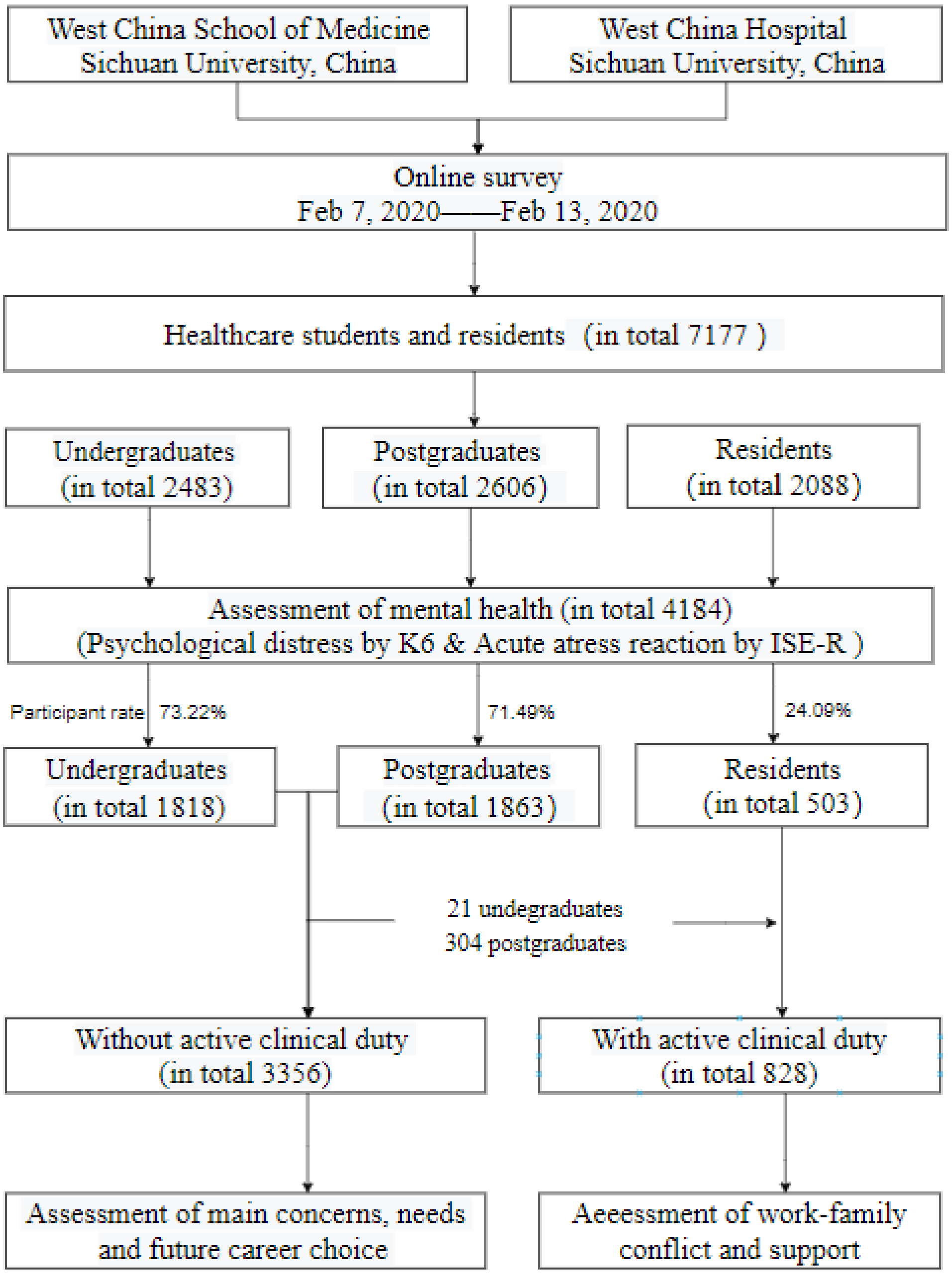

Wang Y, *et al. BMJ Open* 2020; 10:e041671. doi: 10.1136/bmjopen-2020-041671

Supplemental Text:

Demographic information

a) Age: ________ years
b) Sex: A male, B female
c) Training stage: A undergraduate, B postgraduate, C residency
d) Training program: A medicine, B medical technology, C nursing
e) Marital status: A married, B unmarried
f) Location: _______ (current)
g) Have you had relatives infected with COVID-19?
A. No B. Yes
h) Are you actively performing clinical duties at this time?
A. No B. Yes

<u>If Yes, go on:</u>

i) Working position: _________ department.
ii) Since the outbreak, have you contacted with COVID-19 patients?
A. Yes B. No
iii) Work-family conflict and support
Since the outbreak,
a) my current job has made it difficult for me to care for my family.
b) family responsibilities have affected my work.
c) I had difficulties in juggling work and family.
d) I can get support from my family.
e) I can get support from my colleagues.
f) I can get support from my leader.
g) The hospital's holiday arrangement is reasonable.
h) The hospital's duty arrangement is reasonable.
i) The hospital has had a flexible policy that allowed me to juggle family and work.

<u>If No, go on:</u>

i) Concerns during the outbreak (Multiple choices)
Under the current circumstances, I am concerned about
a) being infected with the novel coronavirus;
b) my physical health condition;
c) my psychological health;
d) academic performance;
e) my social life and work;
f) my traveling plans;
g) the risk of infection from family members or friends;
h) my personal and family's financial situation;
i) other issues.
ii) Needs during the outbreak (Multiple choices)

Wang Y, *et al. BMJ Open* 2020; 10:e041671. doi: 10.1136/bmjopen-2020-041671

If I were to work during the outbreak, I would need

a) personal protective equipment;
b) social insurance;
c) salary incentives;
d) clinical practice guidance;
e) professional track record;
f) other needs.

iii) The influence on future career choice (single question)

Has the outbreak affected your future career plans? i) Healthcare worker; ii) Medicine-related, but not bedside; iii) Outside of medicine; iv) Indeterminate.

Psychological distress

Since the COVID-19 outbreak,

a) How often did you feel nervous?
b) How often did you feel hopeless?
c) How often did you feel restless or fidgety?
d) How often did you feel so depressed that nothing could cheer you up?
e) How often did you feel that everything was an effort?
f) How often did you feel worthless?

Acute stress reaction

During the past seven days concerning the COVID-19 outbreak, how much have you been distressed or bothered by these difficulties?

a) Any reminder brought back feelings about it.
b) I had trouble staying asleep.
c) Other things kept making me think about it.
d) I felt irritable and angry.
e) I avoided letting myself get upset when I thought about it or reminded me of it.
f) I thought about it when I did not mean to.
g) I felt as if it had not happened or was not real.
h) I stayed away from reminders of it.
i) Pictures about it popped into my mind.
j) I was jumpy and easily startled.
k) I tried not to think about it.
l) I was aware that I still had many feelings about it, but I did not deal with them.
m) My feelings about it were kind of numb.
n) I found myself acting or feeling like I was back at that time.
o) I had trouble falling asleep.
p) I had waves of strong feelings about it.
q) I tried to remove it from my memory.
r) I had trouble concentrating.
s) Reminders of it caused me to have physical reactions, such as sweating, trouble breathing, nausea, or a pounding heart.

t) I had dreams about it.
u) I felt watchful and on-guard.
v) I tried not to talk about it.

Wang Y, *et al. BMJ Open* 2020; 10:e041671. doi: 10.1136/bmjopen-2020-041671
