# Supplementary material for: COVID-19 outbreak–related psychological distress among healthcare trainees: a cross-sectional study in China
Source: BMJ Open. 2020 Oct 20;10(10):e041671. doi: 10.1136/bmjopen-2020-041671 (PMC7577057; doi:10.1136/bmjopen-2020-041671)
Supplement: Author's manuscript [file bmjopen-2020-041671.draft_revisions.pdf]

BMJ Open is committed to open peer review. As part of this commitment we make the peer review history of every article we publish publicly available.

When an article is published we post the peer reviewers' comments and the authors' responses online. We also post the versions of the paper that were used during peer review. These are the versions that the peer review comments apply to.

The versions of the paper that follow are the versions that were submitted during the peer review process. They are not the versions of record or the final published versions. They should not be cited or distributed as the published version of this manuscript.

BMJ Open is an open access journal and the full, final, typeset and author-corrected version of record of the manuscript is available on our site with no access controls, subscription charges or pay-per-view fees (http://bmjopen.bmj.com).

If you have any questions on BMJ Open's open peer review process please email info.bmjopen@bmj.com

BMJ Open

COVID-19 outbreak-related psychological distress among health professional trainees：a cross-sectional study in China

Journal:	*BMJ Open*
Manuscript ID	bmjopen-2020-041671
Article Type:	Original research
Date Submitted by the Author:	16-Jun-2020
Complete List of Authors:	Wang, Yue; Sichuan University, Mental health center<br>Li, Yuchen; Sichuan University, Mental health center; Karolinska Institute, Department of Medical Epidemiology and Biostatistics<br>Jiang, Jingwen; Sichuan University, West China Biomedical Big Data Center<br>Feng, Yuying; Sichuan University, Student Affairs Office<br>Song, Huan; Sichuan University, West China Biomedical Big Data Center; University of Iceland, Center of Public Health Sciences<br>zhang, wei; Sichuan University,<br>Lu, Donghao ; Karolinska Institutet Department of Medical Epidemiology and Biostatistics; Karolinska Institute, Department of Medical Epidemiology and Biostatistics
Keywords:	PSYCHIATRY, MENTAL HEALTH, MEDICAL EDUCATION & TRAINING, INTERNAL MEDICINE, EPIDEMIOLOGY

SCHOLARONE™
Manuscripts

I, the Submitting Author has the right to grant and does grant on behalf of all authors of the Work (as defined in the below author licence), an exclusive licence and/or a non-exclusive licence for contributions from authors who are: i) UK Crown employees; ii) where BMJ has agreed a CC-BY licence shall apply, and/or iii) in accordance with the terms applicable for US Federal Government officers or employees acting as part of their official duties; on a worldwide, perpetual, irrevocable, royalty-free basis to BMJ Publishing Group Ltd ("BMJ") its licensees and where the relevant Journal is co-owned by BMJ to the co-owners of the Journal, to publish the Work in this journal and any other BMJ products and to exploit all rights, as set out in our licence.

The Submitting Author accepts and understands that any supply made under these terms is made by BMJ to the Submitting Author unless you are acting as an employee on behalf of your employer or a postgraduate student of an affiliated institution which is paying any applicable article publishing charge ("APC") for Open Access articles. Where the Submitting Author wishes to make the Work available on an Open Access basis (and intends to pay the relevant APC), the terms of reuse of such Open Access shall be governed by a Creative Commons licence – details of these licences and which Creative Commons licence will apply to this Work are set out in our licence referred to above.

Other than as permitted in any relevant BMJ Author's Self Archiving Policies, I confirm this Work has not been accepted for publication elsewhere, is not being considered for publication elsewhere and does not duplicate material already published. I confirm all authors consent to publication of this Work and authorise the granting of this licence.

For peer review only

COVID-19 outbreak-related psychological distress among health professional trainees：a cross-sectional study in China

Yue Wang[1#]; Yuchen Li[1,4#]; Jingwen Jiang[2]; Yuying Feng[3]; Huan Song[2,5]*; Wei Zhang[1,2]*; Donghao Lu[4,6,7,8];

Authors' institutions

1. Mental Health Center, West China Hospital of Sichuan University, Chengdu, China;

2. West China Biomedical Big Data Center, West China Hospital, Sichuan University, Chengdu, China;

3. Student Affairs Office, West China School of Medicine, Sichuan University, Chengdu, China;

4. Department of Medical Epidemiology and Biostatistics, Karolinska Institute, Stockholm, Sweden;

5. Center of Public Health Sciences, Faculty of Medicine, University of Iceland, Reykjavík, Iceland;

6. Department of Epidemiology, Harvard T.H. Chan School of Public Health, Boston, Massachusetts;

7. Channing Division of Network Medicine, Brigham and Women's Hospital, Harvard Medical School, Boston, Massachusetts;

8. Clinical Research Center for Breast Diseases, West China Hospital, Sichuan University, Chengdu, China;

[#] Yue Wang and Yuchen Li contribute equally to the study.

* Huan Song, and Wei Zhang contribute equally to the study.

For peer review only - http://bmjopen.bmj.com/site/about/guidelines.xhtml

Correspondence: Huan Song, MD, PhD, West China Biomedical Big Data Center, West China Hospital, Sichuan University, Guoxue Lane 37, Chengdu, China (e-mail: songhuan@wchscu.cn) ; or Wei Zhang, MD, West China Biomedical Big Data Center, West China Hospital, Sichuan University, Guoxue Lane 37, Chengdu, China (e-mail: weizhanghx@163.com).

For peer review only

For peer review only - http://bmjopen.bmj.com/site/about/guidelines.xhtml

Abstract

Objectives The COVID-19 outbreak is placing an enormous strain on the healthcare system, health professional trainees, as the future healthcare workforce, may be a vulnerable group. To assess psychological distress among health professional trainees during the COVID-19 outbreak are necessary.

Design, setting and participants A cross-sectional study including 4181 health professional trainees at Sichuan University in China during February 7-13, 2020. Participants were grouped according to training programs (2727 in Medicine, 944 in Medical Technology, and 513 in Nursing) and training stages (1791 undergraduates,1890 postgraduates, and 503 residents).

Main outcomes COVID-19 outbreak-related psychological distress and acute stress reaction (ASR) were assessed by Kessler 6-item Psychological Distress Scale and the Impact of Event Scale-revised, respectively. We estimated odds ratios (ORs) of distress by comparing trainees across different programs and training stages using multivariable logistic regression.

Results Based on our survey, we identified 1150 (30.90%) participants with significant psychological distress and 403 (10.74%) had probable ASR. Compared to trainees of nursing, medicine trainees reported greater burden of psychological distress (OR 1.57, 95% CI 1.24-1.99) during the outbreak. No evident increase was found in trainees of medical technology. Compared to undergraduates, postgraduates or residents in medicine had a higher level of distress (ORs 1.62-1.66), whereas a lower burden endorsed by nursing residents (OR 0.35, 95% CI 0.19-0.63).Importantly, active clinical duty during the outbreak was significantly associated with distress (OR 1.31, 95% CI 1.10-1.57) in trainees, particularly in medicine trainees (OR 1.92, 95% CI 1.53-2.40) and in undergraduates (OR 4.24, 95% CI 1.62-11.80). We did not observe any clear risk pattern for ASR symptoms.

For peer review only

For peer review only - http://bmjopen.bmj.com/site/about/guidelines.xhtml

Conclusions Medicine trainees, particularly those at senior stages or with active clinical duty, are at risk for psychological distress during the COVID-19 outbreak. Stress management may be considered for high-risk health professional trainees.

For peer review only

For peer review only - http://bmjopen.bmj.com/site/about/guidelines.xhtml

Strengths and limitations of this study

- We comprehensively assessed psychological distress among health professional trainees across different programs and training stages during the COVID-19 outbreak.
- To shed light on the impact on their life/work, we also assessed concerns and needs during the outbreak as well as the influence on future career choice among trainees without active clinical duty, and evaluated work-family conflict and support among trainees with clinical duty.
- Our analyses were limited to the cross-sectional study design in a single medical school/teaching hospital, and results should be qualified by the possibility of interpretation constraints of the survey.

For peer review only

Introduction

Globally, the ongoing pandemic of 2019 novel coronavirus disease (COVID-19) has caused 1,991,562 cases and 130,885 deaths as of April 16th, 2020 [1]. Due to a drastic surge of COVID-19 patients, frontline healthcare workers were exposed to high workloads and at high risk for COVID-19. In addition, witnessing unexpected illnesses or deaths can be devastating. All these factors contributed to an elevated mental burden among healthcare workers. Indeed, emerging data indicated that Chinese healthcare workers exposed to COVID-19 suffered unfavorable psychological symptoms, especially among women, nurses, those in Wuhan (the first epicenter), and frontline workers [2]. Despite limited direct contacts with COVID-19 patients, health professional trainees, as the future healthcare workforce, may be a vulnerable group [3]. As the pandemic escalates, many countries are considering or already graduating senior students early to aid the frontline workers. More aggressive approaches have also been proposed, for instance, suspending all medical schools for one year and recruiting medical students to test, track and quarantine COVID-19 [4]. Although many are inspired at these unprecedented times, some, especially the ones without sufficient clinical experience, may be stressed. We therefore aimed to assess the prevalence of psychological distress and acute stress reaction (ASR) among health professional trainees across different programs and training stages in response to the COVID-19 outbreak.

Materials and methods

Study design

We conducted a cross-sectional study of health professional trainees from the West China School of Medicine and West China Hospital, Sichuan University. During February 7-13, 2020, we invited all eligible individuals to participate through online questionnaires regarding

For peer review only - http://bmjopen.bmj.com/site/about/guidelines.xhtml

basic characteristics and COVID-19 outbreak-related mental health (Supplementary Figure 1 Research Flow Chart).

For undergraduates and graduates who were not involved in clinical work, we exclusively asked their main concerns and needs during the COVID-19 epidemic, as well as the impact of such an experience on their future career plan; whereas clinical workers were prompted to a short survey about work-family conflict and support. In total, we included 1818 undergraduates (participation rate 73.22%), 1863 postgraduates (71.49%), and 503 residents (24.12%). This study was approved by the Ethics Committee of the Sichuan University and electronic consent forms were obtained from all participants.

Due to the Chinese Spring Festival, most undergraduate and postgraduate students were at home across the entire country during the COVID-19 outbreak period, while all residents remained in Chengdu, Sichuan Province due to clinical duties. As of 6 February 2020, the total number of confirmed COVID-19 cases was 344 in Sichuan (102 in Chengdu), and 7,226 individuals were under medical observation[5].

Health professional programs and training stages

The health professional training programs in China mainly consist of medicine, medical technology and nursing, which train for future doctors, medical technologists (including medical laboratory technologists, imaging technologists, physical therapists, optometrists, etc.), and nurses. Training stages was classified into undergraduates, postgraduates, and residents. Briefly, all training programs start from undergraduate programs, of which medicine program is 5 years and program of medical technology/nursing is 4 years. After

For peer review only - http://bmjopen.bmj.com/site/about/guidelines.xhtml

that, individuals continue training in postgraduate program (3-6 years) with a primary focus on research, while clinical training could be combined. For those who pursue a career as a clinician, either after the undergraduate or postgraduate program, they enter residency program (3 years for medicine and 2 years for medical Technology/nursing) for supervised clinical work.

As stated above, due to the co-occurrence with the Chinese Spring Festive, individuals at early training stage had a low proportion of clinical work exposure at the time of survey. In addition, in order to protect students without clinical experience, the medical school canceled clinical practice for undergraduates since the outbreak of COVID-19, while few of senior undergraduates that had internship experiences volunteered to stay and support the clinical work The active clinical workers included 503 residents and 325 students (304 postgraduates and 21 undergraduates). To assess the working status of the trainees, we asked all participants “Are you active in clinical duty at this moment” in the survey.

Assessment of outbreak-related psychological distress

We assessed psychological distress and acute stress reaction (ASR) and phrased the questions specific to COVID-19 outbreak.

Psychological distress was assessed by the Chinses version of Kessler 6-item Psychological Distress Scale (K6). It consists of six items about major depression and generalized anxiety disorder and asks respondents how frequently they experienced these symptoms in the past month[6]. Each item has five ordinal options (from 0 = "never" to 4 = "all of the time"), and the total score ranges from 0 to 24. We considered a score ≥5 as clinically significant distress

For peer review only - http://bmjopen.bmj.com/site/about/guidelines.xhtml

according to validation studies in Asian populations [7, 8]. The Cronbach's alpha value was 0.912 in our study, suggesting a good scale reliability.

Acute stress reaction was evaluated by the Chinese version Impact of Event Scale-revised (ISE-R)[9]. It consists of 22 items related to intrusion, avoidance and hyperarousal posttraumatic symptoms and asked subjects how much they distressed or bothered during the past seven days. Responses were based on a 5-point rating scale, ranging from 0 "not at all" to 4 "extremely". Individuals with a score ≥24 is considered as a probable ASR [10, 11]. The Cronbach α value was 0.907 in our study, suggesting a good scale reliability.

Assessment of outbreak impact

To shed light on the impact on their life/work, we assessed concerns and needs during the outbreak as well as the influence on future career choice among trainees without active clinical duty, and evaluated work-family conflict and support among trainees with clinical duty.

Concerns, needs during the outbreak, and the influence on future career choice: to understand the main concerns and needs, we asked "Under the current circumstances, I am concerned about a) being infected with novel coronavirus; b) my physical health condition; c) my psychological health; d) academic performance; e) my social life/work; f) my traveling plan; g) the risk of infection for family members or friends; h) my personal or family financial situation; i) other things"; and "If I were to work during the outbreak, I need a) personal protective equipment; b) social insurance; c) salary incentives; d) clinical practice guidance; e) professional track record; f) others." Multiple choices were allowed for these questions. To

For peer review only - http://bmjopen.bmj.com/site/about/guidelines.xhtml

understand the effects on future career choice, we used one single question “Has the outbreak affected your future career plan?” to assess the impact of COVID-19 outbreak on their career plan.

Work-family conflict and support: The 9-item Chinses version of “Work-Family Conflict and Support” scale was used to investigate work-family conflict, social support, and policy support[12]. Each dimension has three items with three ordinal options (1 = "agree", 2="neutral", and 3 = "disagree").

Statistical Analysis

First, we compared the baseline characteristics of trainees across different programs (i.e., medicine, medical technology, and nursing) using student t-test (for continuous variables) and Chi-square test (for categorial variables). Next, we calculated the prevalence of psychological distress and probable ASR across different training programs, stages, and status of clinical duty. We estimated coefficients (βs) and odds ratios (ORs) of distress and ASR symptom scores using liner regression and probable cases using logistic regression. All models were adjusted for age and sex (male or female), and additionally adjusted for training program when analyzing the status of clinical duty. Furthermore, among participants without active clinical duty, we examined the associations of concerns, needs and future career choice with distress/ASR; for those with active clinical duty, we assessed the associations of family-work conflicts with distress/ASR. All analyses were conducted in R 3.6.1. $P<0.05$ was considered as statistical significance.

Results

For peer review only - http://bmjopen.bmj.com/site/about/guidelines.xhtml

Demographic characteristics

In a total of 4184 trainees, the mean age was 23.41±3.81 years and 64.89% were females. While no confirmed COVID-19 was reported, 31(0.74%) of them had at least one relative being infected. About one-fifth (19.79%) of partipants were active in clinical duty; among then, 74 (8.94%) were working in the front line and 38 (4.59%) had direct contact with COVID-19 (Table 1). Compared with trainees in medical technology/nursing program, medicine trainees were older, and more likely to be postgraduates, males, married, and outside of Hubei Province – the first epicenter ($P<0.05$). At the time of the survey, nurse trainees were more likely to have active clinical duty and work in the front line ($P<0.05$).

Outbreak-related Psychological distress and Acute Stress Reaction

During the outbreak, significant psychological distress was endorsed by 1150 (30.90%) trainees and probable ASR in 403 (10.74%) participants. Compared to nurse training, medicine training was positively associated with distress symptoms (β 0.26, 95% CI 0.15-0.36) and cases of significant distress (OR 1.57, 95% CI 1.24-1.99; Table 2). Similar pattern was found for medical technology training program, although the association with distress cases was not significant. Compared with undergraduates, postgraduates or residents in medicine program had a higher level of distress, whereas a lower burden endorsed by nursing residents. No evident increase was found across training stages in trainees of medical technology. Similar patterns, yet weaker associations, were observed for ASR symptoms and probable cases across training programs as well as training stages within a program, except for lower burden of ASR reported in medicine residents compared to undergraduates.

Moreover, active clinical duty during the outbreak was positively associated with both

For peer review only - http://bmjopen.bmj.com/site/about/guidelines.xhtml

distress symptoms (β 0.15, 95% CI 0.07-0.24) and cases of significant distress (OR 1.31, 95% CI 1.10-1.57; Table 3). Interestingly, the association between active duty and distress was positive in medicine trainees, but negative in nursing trainees. The association was slightly stronger in undergraduates than that in postgraduates. However, active clinical duty was negatively associated with ASR symptoms (β -0.08, 95% CI -0.17, -0.00), and except for that, no significant association across training program and stages were found for ASR.

Outbreak impact among vulnerable trainees

Among trainees without active clinical duty during the outbreak, individuals who showed significant distress were most concerned of mental health (OR 2.41, 95% CI 1.91-3.05; Table 4) and strongly demanded personal protective equipment if they were to work during the outbreak (OR 1.50, 95% CI 1.07-2.15). Moreover, they were more likely to consider future career outside of medicine (OR 2.91, 95% CI 1.79-4.73). Similar pattern was noted for ASR, except for concerns of academic performance, travel plans, personal/family financial situation and needs of personal protective equipment, which only associated with psychological distress.

Among trainees with active clinical duty, individuals with distress were more likely to report work-family conflicts (ORs 2.03-2.77; Table 5). By contrast, adequate social support (ORs 0.40-0.48) or reasonable work arrangements (ORs 0.38-0.43) were associated with lower psychological distress. Similar pattern was found for ASR.

Discussion

In this large-scale cross-sectional study, we found that psychological distress is common

For peer review only - http://bmjopen.bmj.com/site/about/guidelines.xhtml

among health professional trainees during the COVID-19 outbreak. Moreover, medicine trainees, particularly those at senior stages or with active clinical duty, were at higher risk for psychological distress, compared to those in other training programs or at the earlier training stage. Concerns of mental health condition was strongly correlated with psychological distress among trainees with no clinical duty, whereas work-family conflicts highly concern distressed trainees with active clinical duty.

The COVID-19 outbreak is placing an enormous strain on the healthcare system, and medical trainees alongside with heath professional workers are all in challenging situation [3]. In line with prior findings among healthcare workers [2, 13, 14], our results suggested that psychological distress were also common among health professional trainees, especially the ones in medicine training program. Conversely, several studies reported that nurses providing care to COVID-19 confirmed or suspected patients, had a greater mental health burden than doctors [2, 15]. Given that only a small proportion of our participants (4.59%) had direct contact with COVID-19 patients, these diverse estimates do not necessarily invalidate each other.

Another important finding is the association of advanced training stage and active clinical duty with elevated level of psychological distress. Academic pressure, workload as well as financial burden can increase with the level of training, which could consequently increase the mental vulnerability of senior trainees and ultimately contribute to their negative mental outcomes [16-20]. Moreover, during the COVID-19 outbreak, to deal with the considerable workforce shortage in healthcare system, senior medical students and residents have been encouraged to aid clinical work [21, 22]. The overwhelming workload and high risk of exposure to COVID-19 may add further burden to mental health of this specific population. Indeed,

For peer review only

For peer review only - http://bmjopen.bmj.com/site/about/guidelines.xhtml

across different training stages, we consistently observed a higher risk of psychological distress among individuals who were involved in active clinical duties during the outbreak.

ASR often develop following the direct exposure to traumatic events, such as experiencing the outbreak in the epicenter [23] and directly diagnosing/treating COVID-19 confirmed patients [2, 23]. However, only a few of our participants were quarantined in the epicenter or had direct contact with COVID-19 patients at the time of the survey. The risk of ASR was therefore relatively low and the difference between training programs/stages were small. Interestingly, we found active clinical duty trainees had less ASR symptoms during the outbreak.

Our findings suggested that being infected by COVID-19 was the leading concern in health professional trainees, followed by concerns of mental health, with regards to the risk of psychological distress and ASR. In addition, adequate personal protective equipment and salary incentives may help reduce the level of psychological distress. This is in line with the previous finding that family income stability was a protective factor for medical college students to against anxiety [24]. Moreover, among clinical workers, work-family conflicts were positively associated with psychological distress, whilst we observed reversed association for the social supports. Altogether, in order to reduce the possibility of psychological distress among healthcare trainees, adequate personal protection, timely psychological intervention, stable financial situation, and strong family and social support may be the key things to be considered. Of note, the proper leadership including active participation in outbreak preparedness and making reasonable work arrangements could also alleviate the emotional strain in healthcare workers [25], suggesting the importance of working polices to health

For peer review only - http://bmjopen.bmj.com/site/about/guidelines.xhtml

professional trainees.

Studies have implied that experiencing psychological distress during the training stage may lead to an altered career path [26-28]. This is consistent with our finding that, health professional trainees who decided to work on non-medical fields in the future tend to suffer higher level of psychological distress, compared to the ones determined to continue. During the COVID-19 outbreak, health professional trainees may be mentally vulnerable to the crisis, because of being knowledgeable about medicine which made them more aware of danger at the early stage of outbreak. Meanwhile, the epidemic represents an extreme situation that ´doctor´ was considered as a demanding job with social responsibilities, which might scare or inspire medical trainees, especially the ones without clinical experience. It is therefore interpretable that this crisis also has influenced the choice of future career path among this population.

Limitations

Our study has several limitations. First, the response rate was low in residents and the non-participants were more likely to be the ones with severe stress. The participation rate of undergraduate/postgraduate trainees was satisfiable (73.22% and 71.49% respectively). Such selection is less likely to entirely explain our findings. Second, we only measured distress symptoms once in the early phase of the outbreak. As the symptoms may change over time [29-32], longitudinal studies are needed in future. Third, although the effects of age, sex, training programs and training stages were adjusted in the data analysis, residual confounding remains because additional confounding factors were inapplicable (i.e. marital status, current location, working position, etc.) or not collected (i.e. social economic status). Last, although the trainees came from all parts of the country, our study is based on a single medical

For peer review only - http://bmjopen.bmj.com/site/about/guidelines.xhtml

school/teaching hospital. Further studies from independent populations are warranted.

Conclusions

Our findings suggest that psychological distress in response to the COVID-19 outbreak is common among health professional trainees in China. Medicine trainees, particularly those at senior stages or with active clinical duty, were at higher risk for psychological distress. Stress management may be provided for high-risk health professional trainees during the outbreak, particularly if and when accelerating to join the frontline workforce.

Acknowledgments: The authors thank Lie Zhang (West China School of Medicine of Sichuan University, Chengdu, China) for coordinating the data collection; Fenfen Ge (West China Hospital of Sichuan University, Chengdu, China), Ting Liu (West China Hospital of Sichuan University, Chengdu, China) and Xiao Liao (Southwest University, Chongqing, China) for data collection. Mr Zhang, Miss Ge, Miss Liu and Miss Liao have no conflicts of interest to declare. We also thank the participating students at the West China School of Medicine.

Contributors: Drs Zhang, Lu and Song had full access to all of the data in the study and take responsibility for the integrity of the data and the accuracy of the data analysis. Wang and Li contributed equally to the work.

Concept and design: Wang, Li, Zhang.

Acquisition, analysis, or interpretation of data: Wang, Li, Song, Feng.

Drafting of the manuscript: Wang, Li, Song, Lu.

Critical revision of the manuscript for important intellectual content: all authors.

For peer review only - http://bmjopen.bmj.com/site/about/guidelines.xhtml

Statistical analysis: Wang, Jiang.

Obtained funding: Zhang, Song, Lu.

Administrative, technical, or material support: Zhang, Song, Lu, Feng.

Supervision: Zhang, Song, Lu.

All authors have contributed significantly to this work and have met the qualification of authorship.

Funding: This research was supported by the National Natural Science Foundation of China (No. 81971262 to Dr. Song, No. 81801359 to Dr. Lu), Swedish Research Council (No. 2018-00648 to Dr. Lu), West China Hospital COVID-19 Epidemic Science and Technology Project (No. HX-2019-nCoV-014 to Dr. Song, No. HX-2019-nCoV-019 to Dr. Zhang), and Sichuan University Emergency Grant (No. 2020scunCoVyingji1002 to Dr. Song, No. 2020scunCoVyingji1005 to Dr. Zhang).

Competing interests: All authors declare no competing interests.

Patient and public involvement: Patients and/or the public were not involved in the design, or conduct, or reporting, or dissemination plans of this research.

Ethics approval: This study was approved by the Ethics Committee of the Sichuan University.

Data availability statement: The data that support the findings of this study are available from the corresponding author, WZ, upon reasonable request.

For peer review only

For peer review only - http://bmjopen.bmj.com/site/about/guidelines.xhtml

Reference

1. WHO. World Health Organization: Coronavirus disease (COVID-19) outbreak situation. World Health Organization, 2020.
2. Lai J, Ma S, Wang Y, Cai Z, Hu J, Wei N, et al. Factors Associated With Mental Health Outcomes Among Health Care Workers Exposed to Coronavirus Disease 2019. JAMA Network Open. 2020; 3(3): e203976-e.
3. Baker DM, Bhatia S, Brown S, Cambridge W, Kamarajah SK, A K, et al. Medical student involvement in the COVID-19 response. Lancet. 2020; 395(10232): 1254.
4. Bauchner H, Sharfstein J. A Bold Response to the COVID-19 Pandemic: Medical Students, National Service, and Public Health. JAMA. 2020.
5. Health Commission of Sichuan Province C. The latest situation of new coronavirus pneumonia in Sichun province (released on February 7, 2020). (ed HCoS Province). Available from: https://dwz.cn/Y1p9dhTl, 2020.
6. Kang Y-k, Guo W-J, Xu H, Chen Y-h, Li X-j, Tan Z-p, et al. The 6-item Kessler psychological distress scale to survey serious mental illness among Chinese undergraduates: Psychometric properties and prevalence estimate. Comprehensive Psychiatry. 2015; 63.
7. Furukawa TA, Kawakami N, Saitoh M, Ono Y, Nakane Y, Nakamura Y, et al. The performance of the Japanese version of the K6 and K10 in the World Mental Health Survey Japan. International Journal of Methods in Psychiatric Research. 2008; 17(3): 152-8.
8. Kessler RC, Green JG, Gruber MJ, Sampson NA, Bromet E, Cuitan M, et al. Screening for serious mental illness in the general population with the K6 screening scale: results from the WHO World Mental Health (WMH) survey initiative (vol 19, pg 4, 2010). International Journal of Methods in Psychiatric Research. 2011; 20(1): 62-.
9. Wu KK, Chan KS. The development of the Chinese version of Impact of Event Scale--Revised (CIES-R). (0933-7954 (Print)).
10. Lai YH, Lee YH, Chen YR, Hung YJ. Psychometric Testing of the Impact of Event Scale-Revised (IES-R) Chinese Version and Current Status in Three Major Types of Cancer Survivors in Taiwan. Psycho-Oncology. 2014; 23: 156-.
11. Imsiragic AS, Begic D, Martic-Biocina S. Acute Stress and Depression 3 Days after Vaginal Delivery - Observational, Comparative Study. Collegium Antropologicum. 2009; 33(2): 521-7.
12. HUANG D, YIN W, ZHANG Y, WANG P, SUN K. Effects of Social and Policy Supports on Work-family Conflict in Physicians. Chinese Mental Health Journal. 2009; 23(01): 27-32.
13. Liu C, Yang Y-z, Zhang XM, Xu X, Dou Q-L, Zhang W-W. The prevalence and influencing factors for anxiety in medical workers fighting COVID-19 in China: A cross-sectional survey. medRxiv. 2020: 2020.03.05.20032003.
14. Zhang W-R, Wang K, Yin L, Zhao W-F, Xue Q, Peng M, et al. Mental Health and Psychosocial

For peer review only - http://bmjopen.bmj.com/site/about/guidelines.xhtml

Problems of Medical Health Workers during the COVID-19 Epidemic in China. Psychotherapy and psychosomatics. 2020: 1-9.

15. Liu ZR, Han B, Jiang RM, Huang YQ, Ma C, Wen J, et al. Mental Health Status of Doctors and Nurses During COVID-19 Epidemic in China (3/4/2020). Available from: http://dxdoiorg/102139/ssrn3551329.

16. Dyrbye L, Thomas M, Shanafelt T. Medical Student Distress: Causes, Consequences, and Proposed Solutions. Mayo Clinic proceedings Mayo Clinic. 2006; 80: 1613-22.

17. Chunming WM, Harrison R, MacIntyre R, Travaglia J, Balasooriya C. Burnout in medical students: a systematic review of experiences in Chinese medical schools. BMC Medical Education. 2017; 17(1): 217.

18. Levey RE. Sources of stress for residents and recommendations for programs to assist them. Acad Med. 2001; 76(2): 142-50.

19. Dyrbye LN, Thomas MR, Massie FS, Power DV, Eacker A, Harper W, et al. Burnout and suicidal ideation among U.S. medical students. Ann Intern Med. 2008; 149(5): 334-41.

20. Moffat KJ, McConnachie A Fau - Ross S, Ross S Fau - Morrison JM, Morrison JM. First year medical student stress and coping in a problem-based learning medical curriculum. (0308-0110 (Print)).

21. Stokes DC. Senior Medical Students in the COVID-19 Response: An Opportunity to Be Proactive. Academic emergency medicine : official journal of the Society for Academic Emergency Medicine. 2020; 27(4): 343-5.

22. Services CfMM. Teaching Hospitals, Teaching Physicians and Medical Residents: CMS Flexibilities to Fight COVID-19. (ed CfMM Services): 2020.

23. Sun L, Sun Z, Wu L, Zhu Z, Zhang F, Shang Z, et al. Prevalence and Risk Factors of Acute Posttraumatic Stress Symptoms during the COVID-19 Outbreak in Wuhan, China. medRxiv. 2020: 2020.03.06.20032425.

24. Cao W, Fang Z, Hou G, Han M, Xu X, Dong J, et al. The psychological impact of the COVID-19 epidemic on college students in China. Psychiatry research. 2020; 287: 112934.

25. Mason DJ, Friese CR. Protecting health care workers against COVID-19—and being prepared for future pandemics. JAMA Health Forum. 2020; 1(3):e200353.

26. Melincavage SM. Student nurses' experiences of anxiety in the clinical setting. (1532-2793 (Electronic)).

27. Hawkins M. 2017 Final Year Medical Residents Survey. 2018.

28. Scharer S, Freitag A. Physicians' exodus: why medical graduates leave Austria or do not work in clinical practice. (1613-7671 (Electronic)).

29. Leung GM, Ho L-M, Chan SKK, Ho S-Y, Bacon-Shone J, Choy RYL, et al. Longitudinal assessment of community psychobehavioral responses during and after the 2003 outbreak of severe acute respiratory syndrome in Hong Kong. Clinical infectious diseases : an official publication of the Infectious Diseases Society of America. 2005; 40(12): 1713-20.

30. Pietrzak RH, Tracy M, Galea S, Kilpatrick DG, Ruggiero KJ, Hamblen JL, et al. Resilience in the

For peer review only - http://bmjopen.bmj.com/site/about/guidelines.xhtml

Face of Disaster: Prevalence and Longitudinal Course of Mental Disorders following Hurricane Ike. PLOS ONE. 2012; 7(6): e38964.

31. James PB, Wardle J, Steel A, Adams J. Post-Ebola psychosocial experiences and coping mechanisms among Ebola survivors: a systematic review. Trop Med Int Health. 2019; 24(6): 671-91.

32. Ye Y, Fan F, Li LY, Han QG. Trajectory and predictors of depressive symptoms among adolescent survivors following the Wenchuan earthquake in China: a cohort study. Social Psychiatry and Psychiatric Epidemiology. 2014; 49(6): 943-52.

For peer review only

Table 1 Characteristics of health professional trainees – N (%) or mean (SD).

	All	Medicine	Medical technology	Nursing	P
**Participants, N**	4184	2727 (65.18)	944 (22.56)	513 (12.26)	
**Age, years, mean (SD)**	23.41 (3.81)	24.20 (3.84)	21.90 (3.24)	21.98 (3.36)	<0.01
**Training stage**					<0.01
Undergraduates	1791 (42.81)	940 (34.47)	588 (62.29)	263 (51.27)	
Postgraduates	1890 (45.17)	1662 (60.95)	142 (15.04)	86 (16.76)	
Residents	503 (12.02)	125 (4.58)	214 (22.67)	164 (31.97)	
**Sex**					<0.01
Male	1469 (35.11)	1133 (41.55)	265 (28.07)	71 (13.84)	
Female	2715 (64.89)	1594 (58.45)	679 (71.93)	442 (86.16)	
**Marital status**					<0.01
Married	331 (7.91)	254 (9.31)	48 (5.08)	29 (5.65)	
Unmarried	3853 (92.09)	2473 (90.69)	896 (94.92)	484 (94.35)	
**Location**					0.02
Hubei [a]	67 (1.60)	46 (1.69)	20 (2.12)	1 (0.19)	
Outside Hubei	4117 (98.40)	2681 (98.31)	924 (97.88)	512 (99.81)	
**Relatives with COVID-19**					0.90
No	4153 (99.26)	2706 (99.23)	937 (99.26)	510 (99.42)	
Yes	31 (0.74)	21 (0.77)	7 (0.74)	3 (0.58)	
**Active clinical duty**					<0.01
No	3356 (80.21)	2301 (84.38)	719 (76.17)	336 (65.50)	
Yes	828 (19.79)	426 (15.62)	225 (23.83)	177 (34.50)	
**Working position [b]**					<0.01
Frontline [c]	74 (8.94)	36 (8.54)	5 (2.22)	33 (18.64)	
Second-line	754 (91.06)	390 (91.55)	220 (97.78)	144 (81.36)	
**Contact with COVID-19 [b]**					0.11
Yes	38 (4.59)	24 (5.63)	11 (4.89)	3 (1.69)	
No	790 (95.41)	402 (94.37)	214 (95.11)	174 (98.31)	

a. Hubei Province was the epicenter at the time of the survey.
b. Information was only assessed for participants with activity clinical duty.
c. Frontline working positions was defined as working in departments directly engaging in care for patients with COVID-19, including Emergency, Respiratory, Critical Care Medicine, and Infectious Disease Departments.

For peer review only - http://bmjopen.bmj.com/site/about/guidelines.xhtml

Table 2 Psychological distress and Acute stress reaction among health professional trainees between different training programs and stages.

	Psychological distress [a]				Acute stress reaction [b]			
	Symptoms		Cases		Symptoms		Cases (403)	
	Mean±SD	β (95% CI) [c]	N (%)	OR (95% CI) [c]	Mean±SD	β (95% CI) [c]	N (%)	OR (95% CI) [c]
Training program								
Medicine	0.06±1.05	0.26 (0.15, 0.36)	792 (33.22)	1.57 (1.24, 1.99)	0.01±1.01	0.14 (0.04, 0.24)	256 (10.66)	1.11 (0.80, 1.55)
Medical technology	-0.07±0.92	0.14 (0.03, 0.25)	242 (27.94)	1.26 (0.97, 1.63)	0.03±0.99	0.13 (0.02, 0.24)	96 (11.00)	1.08 (0.76, 1.56)
Nursing	-0.18±0.85	Ref.	116 (24.58)	Ref.	-0.08±0.95	Ref.	51 (10.71)	Ref.
Medicine								
By training stage								
Undergraduates	-0.10±0.99	Ref.	223 (26.05)	Ref.	-0.01±1.06	Ref.	86 (9.92)	Ref.
Postgraduates	0.16±1.08	0.26 (0.13, 0.40)	529 (37.23)	1.66 (1.25, 2.19)	0.02±0.99	0.06 (-0.08, 0.19)	163 (11.39)	1.22 (0.80, 1.87)
Residents	0.10±0.90	0.19 (-0.04, 0.41)	40 (37.38)	1.62 (1.02, 2.56)	-0.07±0.84	-0.06 (-0.28, 0.17)	7 (6.73)	0.65 (0.25, 1.43)
Medical technology								
By training stage								
Undergraduates	-0.07±0.97	Ref.	155 (28.23)	Ref.	0.06±1.05	Ref.	66 (11.98)	Ref.
Postgraduates	-0.02±0.82	-0.14 (-0.39, 0.12)	36 (30.00)	0.83 (0.44, 1.54)	0.01±0.90	-0.09 (-0.36, 0.18)	11 (8.80)	0.60 (0.23, 1.51)
Residents	-0.10±0.84	-0.16 (-0.35, 0.04)	51 (25.89)	0.73 (0.45, 1.16)	-0.06±0.86	-0.15 (-0.35, 0.06)	19 (9.64)	0.70 (0.35, 1.37)
Nursing								
By training stage								
Undergraduates	-0.10±0.90	Ref.	75 (29.88)	Ref.	0.05±1.05	Ref.	32 (12.75)	Ref.
Postgraduates	-0.02±0.96	0.05 (-0.30, 0.40)	21 (29.58)	0.90 (0.35, 2.23)	0.04±0.88	-0.08 (-0.46, 0.30)	8 (10.67)	0.69 (0.17, 2.42)
Residents	-0.39±0.67	-0.31 (-0.51, -0.11)	20 (13.33)	0.35 (0.19, 0.63)	-0.36±0.73	-0.43 (-0.66, -0.21)	11 (7.33)	0.49 (0.21, 1.08)

a. In this analysis, 462 (11.04%) individuals who missed the measure of psychological distress were not included.
b. In this analysis, 433 (10.35%) individuals who missed the measure of acute stress reaction were not included.
c. Estimates were adjusted for age and sex.

For peer review only - http://bmjopen.bmj.com/site/about/guidelines.xhtml

Table 3 Psychological distress and Acute stress reaction among health professional trainees with and without active clinical duty.

	Psychological distress [a]				Acute stress reaction [b]			
	Symptoms		Cases		Symptoms		Cases	
	Mean±SD	β (95% CI) [c]	N (%)	OR (95% CI) [c]	Mean±SD	β (95% CI) [c]	N (%)	OR (95% CI) [c]
Active clinical duty								
No	-0.03±0.99	Ref.	882 (29.73)	Ref.	0.02±1.02	Ref.	326 (10.84)	Ref.
Yes	0.12±1.02	0.15 (0.07, 0.24)	268 (35.50)	1.31 (1.10, 1.57)	-0.07±0.92	-0.08 (-0.17, 0.00)	77 (10.36)	0.96 (0.73, 1.26)
By training program								
Medicine								
No duty	-0.00±1.02	Ref.	609 (30.53)	Ref.	0.01±1.02	Ref.	212 (10.47)	Ref.
With duty	0.39±1.10	0.37 (0.26, 0.49)	183 (47.04)	1.92 (1.53, 2.40)	-0.00±0.98	-0.001 (-0.12, 0.11)	44 (11.67)	1.14 (0.79, 1.60)
Medical technology								
No duty	-0.08±0.94	Ref.	184 (27.84)	Ref.	0.04±1.02	Ref.	76 (11.38)	Ref.
With duty	-0.06±0.86	-0.04 (-0.20, 0.12)	58 (28.29)	0.94 (0.64, 1.38)	-0.03±0.88	-0.08 (-0.25, 0.09)	20 (9.76)	0.87 (0.49, 1.53)
Nursing								
No duty	-0.10±0.90	Ref.	89 (28.62)	Ref.	0.03±1.01	Ref.	38 (12.06)	Ref.
With duty	-0.33±0.75	-0.24 (-0.41, -0.07)	27 (16.77)	0.49 (0.29, 0.80)	-0.30±0.78	-0.34 (-0.53, -0.15)	13 (8.07)	0.62 (0.30, 1.21)
By training stage								
Undergraduates								
No duty	-0.10±0.96	Ref.	442 (26.98)	Ref.	0.02±1.05	Ref.	181 (10.96)	Ref.
With duty	0.54±1.18	0.62 (0.17, 1.08)	11 (61.11)	4.24 (1.62, 11.80)	-0.05±1.16	0.01 (-0.48, 0.51)	3 (16.67)	1.75 (0.39, 5.59)
Postgraduates								
No duty	0.06±1.02	Ref.	440 (33.11)	Ref.	0.01±0.97	Ref.	145 (10.69)	Ref.
With duty	0.52±1.14	0.47 (0.34, 0.61)	146 (51.59)	2.21 (1.69, 2.87)	0.06±1.03	0.07 (-0.06, 0.20)	37 (13.50)	1.32 (0.88, 1.93)
Residents								
No duty	-	Ref.	-	Ref.	-	Ref.	-	Ref.
With duty	-0.15±0.82	-	111 (24.45)	-	-0.16±0.82	-	37 (8.20)	-

a. In this analysis, 462 (11.04%) individuals who missed the measure of psychological distress were not included.
b. In this analysis, 433 (10.35%) individuals who missed the measure of acute stress reaction were not included.
c. Estimates were adjusted for age, sex, and training programs.

For peer review only - http://bmjopen.bmj.com/site/about/guidelines.xhtml

Table 4. Associations of concerns and needs during COVID-19 outbreaks with psychological distress and Acute stress reaction among health professional trainees without active clinical duty.

	Psychological distress [a]			Acute stress reaction [b]		
	No (n=2085)	Yes (n=882)		No (n=2682)	Yes (n=326)	
	N (%)	N (%)	OR (95% CI) [c]	N (%)	N (%)	OR (95% CI) [c]
**Concerns**						
Being infected with novel coronavirus	1298 (62.25)	644 (73.02)	1.42 (1.09, 1.60)	1713 (63.87)	256 (78.53)	1.42 (1.05, 1.95)
Physical health condition	458 (21.97)	316 (35.83)	1.32 (1.16, 1.73)	630 (23.49)	154 (47.24)	1.80 (1.37, 2.38)
Psychological health	189 (9.06)	215 (24.38)	2.41 (1.91, 3.05)	294 (10.69)	120 (36.81)	3.26 (2.44, 4.33)
Academic performance	1195 (57.31)	609 (69.05)	1.30 (1.09, 1.56)	1606 (59.88)	220 (67.48)	0.99 (0.75, 1.29)
Social life/work	383 (18.37)	212 (24.04)	1.19 (0.95, 1.48)	505 (18.83)	99 (30.37)	1.37 (1.01, 1.86)
Traveling plan	141 (6.76)	102 (11.56)	1.36 (1.01, 1.82)	201 (7.49)	46 (14.11)	1.21 0.81, 1.78)
Family members or friends being infected with novel coronavirus	1297 (62.21)	599 (67.91)	0.94 (0.78, 1.13)	1688 (62.94)	231 (70.86)	0.91 (0.69, 1.21)
Personal or family financial situations	249 (11.94)	171 (19.39)	1.28 (1.01, 1.62)	353 (13.16)	75 (23.01)	1.18 (0.85, 1.61)
**Needs**						
Personal protective equipment	1907 (91.46)	837 (94.90)	1.50 (1.07, 2.15)	2485 (92.65)	299 (91.72)	0.73 (0.48, 1.17)
Social insurance	1819 (87.24)	808 (91.61)	1.32 (0.99, 1.77)	2371 (88.40)	292 (89.57)	0.88 (0.59, 1.35)
Salary incentives	1386 (66.47)	646 (73.24)	1.37 (1.13, 1.67)	1809 (67.45)	254 (77.91)	1.79 (1.33, 2.44)
Clinical practice guidance	1317 (63.17)	549 (62.34)	0.89 (0.74, 1.08)	1672 (62.34)	219 (67.18)	1.13 (0.85, 1.51)
Professional track record	1213 (58.18)	516 (58.50)	0.91 (0.75, 1.11)	1551 (57.83)	199 (61.04)	0.92 (0.69, 1.23)
**Future career choice**						
Healthcare worker	1256 (60.24)	446 (50.57)	Ref.	1587 (59.17)	140 (42.94)	Ref.
Medicine-related, but not bedside	618 (29.64)	271 (30.73)	1.26 (1.05, 1.52)	791 (29.49)	109 (33.44)	1.54 (1.17, 2.03)
Outside of medicine	36 (1.73)	35 (3.97)	2.91 (1.79, 4.73)	51 (1.90)	19 (5.83)	4.17 (2.33, 7.21)
Indeterminate	175 (8.39)	130 (14.74)	2.13 (1.65, 2.75)	253 (9.43)	58 (17.79)	2.58 (1.83, 3.59)

For peer review only - http://bmjopen.bmj.com/site/about/guidelines.xhtml

a. In this analysis, 389 (11.59%) individuals who missed the measure of psychological distress were not included.
b. In this analysis, 348 (10.37%) individuals who missed the measure of acute stress reaction were not included.
c. Estimates were adjusted for age, sex, training programs, and training stage

For peer review only

For peer review only - http://bmjopen.bmj.com/site/about/guidelines.xhtml

Table 5 Associations of family-work conflicts during COVID-19 outbreak with psychological distress and Acute stress reaction among health professional trainees with active clinical duty.

	**Psychological distress[b]**			**Acute stress reaction[a]**		
	No (n=487)	Yes (n=268)		No (n=666)	Yes (n=77)	
	N (%)	N (%)	OR (95% CI) [c]	N (%)	N (%)	OR (95% CI) [c]
**Work-family conflict**						
**Difficult to care for family due to work**						
Agree	122 (18.32)	28 (36.36)	2.59 (1.72, 3.91)	59 (12.11)	93 (34.70)	1.85 (1.07. 3.16)
Neutral	331 (49.70)	42 (54.55)	Ref.	249 (5.13)	135 (50.37)	Ref.
Disagree	213 (31.98)	7 (9.09)	0.46 (0.30, 0.69)	179 (36.76)	40 (14.93)	0.25 (0.10, 0.54)
**Family responsibilities affected work**						
Agree	37 (5.56)	14 (18.18)	2.03 (1.08, 3.91)	19 (3.90)	33 (12.31)	2.30 (1.11, 4.60)
Neutral	265 (39.79)	44 (57.14)	Ref.	172 (35.32)	145 (54.10)	Ref.
Disagree	364 (54.65)	19 (24.88)	0.39 (0.28, 0.55)	296 (60.78)	90 (33.58)	0.31 (0.17, 0.55)
**Difficulties in juggling work and family**						
Agree	36 (5.41)	14 (18.18)	2.77 (1.41, 5.70)	14 (2.87)	37 (13.81)	2.54 (1.20, 5.25)
Neutral	246 (36.94)	43 (55.84)	Ref.	155 (31.83)	140 (52.24)	Ref.
Disagree	384 (57.66)	20 (25.97)	0.37 (0.26, 0.52)	318 (65.30)	91 (33.96)	0.28 (0.16, 0.50)
**Social support**						
**Support from family**						
Agree	466 (69.97)	35 (45.45)	0.48 (0.33, 0.68)	359 (73.72)	148 (55.22)	0.41 (0.25, 0.70)
Neutral	166 (24.92)	31 (40.26)	Ref.	107 (21.97)	95 (35.45)	Ref.
Disagree	34 (5.11)	11 (14.29)	1.19 (0.60, 2.37)	21 (4.31)	25 (9.33)	1.64 (0.72, 3.54)
**Support from colleagues**						
Agree	545 (81.83)	44 (57.14)	0.40 (0.27, 0.60)	414 (85.01)	181 (67.54)	0.31 (0.18, 0.53)
Neutral	110 (16.52)	30 (38.96)	Ref.	65 (13.35)	81 (30.22)	Ref.

For peer review only - http://bmjopen.bmj.com/site/about/guidelines.xhtml

	Psychological distress[b]			Acute stress reaction[a]		
	No (n=487)	Yes (n=268)		No (n=666)	Yes (n=77)	
Disagree	11 (1.65)	3 (3.90)	0.50 (0.15, 1.62)	8 (1.64)	6 (2.24)	0.99 (0.21, 3.48)
**Support from supervisors**						
Agree	505 (75.83)	30 (38.96)	0.41 (0.28, 0.58)	389 (79.98)	150 (55.97)	0.21 (0.12, 0.37)
Neutral	146 (21.92)	39 (50.65)	Ref.	87 (17.86)	105 (39.18)	Ref.
Disagree	15 (2.25)	8 (10.39)	0.84 (0.35, 2.08)	11 (2.26)	13 (4.85)	2.22 (0.82, 5.72)
**Policy support**						
**Reasonable holiday arrangement**						
Agree	463 (69.52)	28 (36.36)	0.41 (0.28, 0.60)	369 (75.77)	126 (47.01)	0.26 (0.15, 0.46)
Neutral	156 (23.42)	34 (44.16)	Ref.	94 (19.30)	102 (38.06)	Ref.
Disagree	47 (7.06)	15 (19.48)	1.34 (0.73, 2.47)	24 (4.93)	40 (14.93)	1.44 (0.69, 2.91)
**Reasonable duty arrangement**						
Agree	468 (70.27)	29 (37.66)	0.43 (0.30, 0.63)	371 (76.18)	129 (48.13)	0.26 (0.15, 0.45)
Neutral	163 (24.47)	37 (48.05)	Ref.	100 (20.53)	106 (39.55)	Ref.
Disagree	35 (5.26)	11 (14.29)	1.70 (0.87, 3.41)	16 (3.29)	33 (12.31)	1.28 (0.57, 2.74)
**Flexible policies to balance family and work**						
Agree	438 (65.77)	25 (32.47)	0.38 (0.27, 0.55)	356 (73.10)	109 (40.67)	0.28 (0.16, 0.51)
Neutral	200 (30.03)	36 (46.75)	Ref.	120 (24.64)	123 (45.90)	Ref.
Disagree	28 (4.20)	16 (20.78)	3.14 (1.54, 6.84)	11 (2.26)	36 (13.43)	3.35 (1.60, 6.90)

a. In this analysis, 85 (10.27%) individuals who missed the measure of psychological distress were not included.
b. In this analysis, 73 (8.82%) individuals who missed the measure of acute stress reaction were not included.
c. Adjusted for age, sex, training programs, and training stages.

For peer review only

For peer review only - http://bmjopen.bmj.com/site/about/guidelines.xhtml

Figure 1 Research Flow Chart

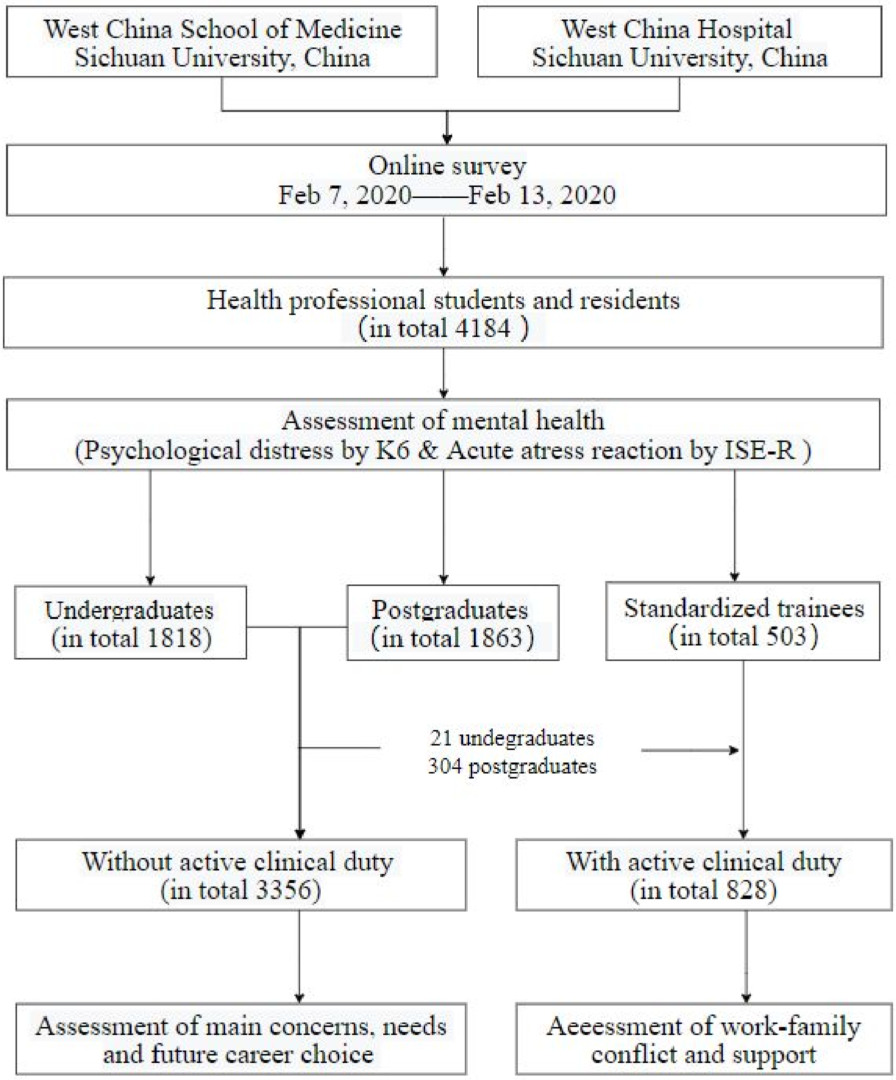

STROBE Statement—Checklist of items that should be included in reports of ***cross-sectional studies***

	Item No	Recommendation	Page No
**Title and abstract**	1	(*a*) Indicate the study's design with a commonly used term in the title or the abstract	1
		(*b*) Provide in the abstract an informative and balanced summary of what was done and what was found	3
**Introduction**			
Background/rationale	2	Explain the scientific background and rationale for the investigation being reported	6
Objectives	3	State specific objectives, including any prespecified hypotheses	6
**Methods**			
Study design	4	Present key elements of study design early in the paper	6
Setting	5	Describe the setting, locations, and relevant dates, including periods of recruitment, exposure, follow-up, and data collection	6-7
Participants	6	(*a*) Give the eligibility criteria, and the sources and methods of selection of participants	7
Variables	7	Clearly define all outcomes, exposures, predictors, potential confounders, and effect modifiers. Give diagnostic criteria, if applicable	8-10
Data sources/ measurement	8*	For each variable of interest, give sources of data and details of methods of assessment (measurement). Describe comparability of assessment methods if there is more than one group	8-10
Bias	9	Describe any efforts to address potential sources of bias	15
Study size	10	Explain how the study size was arrived at	7
Quantitative variables	11	Explain how quantitative variables were handled in the analyses. If applicable, describe which groupings were chosen and why	8-10
Statistical methods	12	(*a*) Describe all statistical methods, including those used to control for confounding	10
		(*b*) Describe any methods used to examine subgroups and interactions	Not applicable
		(*c*) Explain how missing data were addressed	20-26
		(*d*) If applicable, describe analytical methods taking account of sampling strategy	Not applicable
		(*e*) Describe any sensitivity analyses	Not applicable
**Results**			
Participants	13*	(a) Report numbers of individuals at each stage of study—eg numbers potentially eligible, examined for eligibility, confirmed eligible, included in the study, completing follow-up, and analysed	Supplementary
		(b) Give reasons for non-participation at each stage	Not applicable
		(c) Consider use of a flow diagram	Supplementary
Descriptive data	14*	(a) Give characteristics of study participants (eg demographic, clinical, social) and information on exposures and potential confounders	10-11,20
		(b) Indicate number of participants with missing data for each variable of interest	20-26

For peer review only - http://bmjopen.bmj.com/site/about/guidelines.xhtml

Outcome data	15*	Report numbers of outcome events or summary measures	10-11
Main results	16	(*a*) Give unadjusted estimates and, if applicable, confounder-adjusted estimates and their precision (eg, 95% confidence interval). Make clear which confounders were adjusted for and why they were included	20-26
		(*b*) Report category boundaries when continuous variables were categorized	8-9
		(*c*) If relevant, consider translating estimates of relative risk into absolute risk for a meaningful time period	Not appliable
Other analyses	17	Report other analyses done—eg analyses of subgroups and interactions, and sensitivity analyses	Not appliable
**Discussion**			
Key results	18	Summarise key results with reference to study objectives	12
Limitations	19	Discuss limitations of the study, taking into account sources of potential bias or imprecision. Discuss both direction and magnitude of any potential bias	15
Interpretation	20	Give a cautious overall interpretation of results considering objectives, limitations, multiplicity of analyses, results from similar studies, and other relevant evidence	13-15
Generalisability	21	Discuss the generalisability (external validity) of the study results	15
**Other information**			
Funding	22	Give the source of funding and the role of the funders for the present study and, if applicable, for the original study on which the present article is based	16-17

*Give information separately for exposed and unexposed groups.

Note: An Explanation and Elaboration article discusses each checklist item and gives methodological background and published examples of transparent reporting. The STROBE checklist is best used in conjunction with this article (freely available on the Web sites of PLoS Medicine at http://www.plosmedicine.org/, Annals of Internal Medicine at http://www.annals.org/, and Epidemiology at http://www.epidem.com/). Information on the STROBE Initiative is available at www.strobe-statement.org.

For peer review only

BMJ Open

COVID-19 outbreak-related psychological distress among healthcare trainees: a cross-sectional study in China

Journal:	*BMJ Open*
Manuscript ID	bmjopen-2020-041671.R1
Article Type:	Original research
Date Submitted by the Author:	01-Sep-2020
Complete List of Authors:	Wang, Yue; Sichuan University, Mental health center<br>Li, Yuchen; Sichuan University, Mental health center; Karolinska Institute, Department of Medical Epidemiology and Biostatistics<br>Jiang, Jingwen; Sichuan University, West China Biomedical Big Data Center<br>Feng, Yuying; Sichuan University, Student Affairs Office<br>Song, Huan; Sichuan University, West China Biomedical Big Data Center; University of Iceland, Center of Public Health Sciences<br>Zhang, Wei; Sichuan University,<br>Lu, Donghao ; Karolinska Institutet Department of Medical Epidemiology and Biostatistics; Karolinska Institute, Department of Medical Epidemiology and Biostatistics
**Primary Subject Heading**:	Mental health
Secondary Subject Heading:	Epidemiology, Medical education and training
Keywords:	PSYCHIATRY, MENTAL HEALTH, MEDICAL EDUCATION & TRAINING, EPIDEMIOLOGY, COVID-19

SCHOLARONE™
Manuscripts

I, the Submitting Author has the right to grant and does grant on behalf of all authors of the Work (as defined in the below author licence), an exclusive licence and/or a non-exclusive licence for contributions from authors who are: i) UK Crown employees; ii) where BMJ has agreed a CC-BY licence shall apply, and/or iii) in accordance with the terms applicable for US Federal Government officers or employees acting as part of their official duties; on a worldwide, perpetual, irrevocable, royalty-free basis to BMJ Publishing Group Ltd ("BMJ") its licensees and where the relevant Journal is co-owned by BMJ to the co-owners of the Journal, to publish the Work in this journal and any other BMJ products and to exploit all rights, as set out in our licence.

The Submitting Author accepts and understands that any supply made under these terms is made by BMJ to the Submitting Author unless you are acting as an employee on behalf of your employer or a postgraduate student of an affiliated institution which is paying any applicable article publishing charge ("APC") for Open Access articles. Where the Submitting Author wishes to make the Work available on an Open Access basis (and intends to pay the relevant APC), the terms of reuse of such Open Access shall be governed by a Creative Commons licence – details of these licences and which Creative Commons licence will apply to this Work are set out in our licence referred to above.

Other than as permitted in any relevant BMJ Author's Self Archiving Policies, I confirm this Work has not been accepted for publication elsewhere, is not being considered for publication elsewhere and does not duplicate material already published. I confirm all authors consent to publication of this Work and authorise the granting of this licence.

For peer review only

COVID-19 outbreak-related psychological distress among healthcare trainees: a cross-sectional study in China

Yue Wang[1#]; Yuchen Li[1,2#]; Jingwen Jiang[3]; Yuying Feng[4]; Huan Song[3,5]*; Wei Zhang[1,3]*; Donghao Lu[2,6,7,8]

Authors' institutions

1. Mental Health Center, West China Hospital of Sichuan University, Chengdu, China;
2. Department of Medical Epidemiology and Biostatistics, Karolinska Institute, Stockholm, Sweden;
3.West China Biomedical Big Data Center, West China Hospital, Sichuan University, Chengdu, China;
4. Student Affairs Office, West China School of Medicine, Sichuan University, Chengdu, China;
5. Center of Public Health Sciences, Faculty of Medicine, University of Iceland, Reykjavík, Iceland;
6. Department of Epidemiology, Harvard T.H. Chan School of Public Health, Boston, Massachusetts;
7. Channing Division of Network Medicine, Brigham and Women's Hospital, Harvard Medical School, Boston, Massachusetts;
8. Clinical Research Center for Breast Diseases, West China Hospital, Sichuan University, Chengdu, China;

[#] Yue Wang and Yuchen Li contribute equally to the study.

* Huan Song, and Wei Zhang contribute equally to the study.

For peer review only

For peer review only - http://bmjopen.bmj.com/site/about/guidelines.xhtml

Correspondence: Huan Song, MD, PhD, West China Biomedical Big Data Center, West China Hospital, Sichuan University, Guoxue Lane 37, Chengdu, China (e-mail: songhuan@wchscu.cn); or Wei Zhang, MD, West China Biomedical Big Data Center, West China Hospital, Sichuan University, Guoxue Lane 37, Chengdu, China (e-mail: weizhanghx@163.com).

For peer review only

For peer review only - http://bmjopen.bmj.com/site/about/guidelines.xhtml

Abstract

Objectives The COVID-19 outbreak has caused enormous strain on healthcare systems, and healthcare trainees, which comprise the future healthcare workforce, may be a vulnerable group. It is essential to assess the psychological distress experienced by healthcare trainees during the COVID-19 outbreak.

Design, setting, and participants A cross-sectional study with 4184 healthcare trainees at Sichuan University in China was implemented during February 7-13, 2020. Participants were grouped by training program (medicine, medical technology, and nursing) and training stages (undergraduate, postgraduate, and residency).

Main outcomes COVID-19-related psychological distress and acute stress reaction (ASR) were assessed using the Kessler 6-item Psychological Distress Scale and the Impact of Event Scale-Revised, respectively. We estimated the odds ratios (ORs) of distress by comparing trainees across programs and training stages using multivariable logistic regression.

Results Significant psychological distress was found in 1150 (30.90%) participants and probable ASR in 403 (10.74%). Compared to the nursing trainees, the medical trainees (OR 1.54, 95% CI 1.22 - 1.95) reported a higher burden of psychological distress during the outbreak, whilst the medical technology trainees (OR 1.25, 95%CI 0.97 - 1.62) reported similar symptom scores. Postgraduates (OR 1.55, 95%CI 1.16 - 2.08) in medicine had higher levels of distress than their undergraduate counterparts did, whereas the nursing residents (OR 0.38, 95%CI 0.20 - 0.71) reported a lower burden than did nursing undergraduates. A positive association was found between having active clinical duties during the outbreak and distress (OR 1.17, 95%CI 0.98 - 1.39), particularly among the medical trainees (OR 1.85, 95%CI 1.47 - 2.33) and undergraduates (OR 4.20, 95%CI 1.61 - 11.70). No clear risk patterns of ASR symptoms were observed.

Conclusions Medical trainees, particularly postgraduates and those with active clinical

For peer review only - http://bmjopen.bmj.com/site/about/guidelines.xhtml

duties, were at risk for psychological distress during the COVID-19 outbreak. Stress management may be considered for high-risk healthcare trainees.

For peer review only - http://bmjopen.bmj.com/site/about/guidelines.xhtml

Strengths and limitations of this study

- We assessed psychological distress among healthcare trainees across different programs and training stages during the COVID-19 outbreak.
- To shed light on the pandemic's impact on trainees' lives and work, we assessed their concerns and needs during the outbreak and their influence on the future career choices of the trainees without active clinical duties; we also evaluated work-family conflict and support among trainees with clinical duties.
- Our analyses were limited by the study's cross-sectional design, its setting in a single medical school and teaching hospital; hence, the results should be interpreted in light of these limitations and the survey's constraints.

For peer review only

For peer review only - http://bmjopen.bmj.com/site/about/guidelines.xhtml

Introduction

The ongoing global pandemic of the 2019 novel coronavirus disease (COVID-19) has caused 1,991,562 cases and 130,885 deaths as of April 16th, 2020[1]. Witnessing an unexpected illness or death, fear of being in direct contact with and infected by patients with COVID-19, and dealing with household financial hardships during the outbreak has increased the mental burden in the general population[2]. These factors have also elevated the mental burden of healthcare trainees and workers[3-5], with frontline workers having heavy workloads and being placed at higher risk for COVID-19, due to the drastic surge in patients with COVID-19. Emerging data indicate that Chinese healthcare workers exposed to COVID-19 have experienced psychological symptoms, especially women, nurses, those in Wuhan (the first epicenter), and frontline workers[6]. Other studies have reported a profound mental impact of the COVID-19 outbreak on healthcare workers globally[3, 5, 7].

Despite their limited direct contact with patients with COVID-19, healthcare trainees are a vulnerable group[8]. As the pandemic escalates, many countries are considering, or have already graduated senior students earlier to assist frontline workers. Other aggressive approaches have been proposed, for instance, suspending all medical school education for one year and recruiting medical students tor testing, tracking, and quarantining patients with COVID-19[9]. Although many trainees are inspired during these unprecedented times, some, especially those without sufficient clinical experience, may experience stress. Nevertheless, the psychological state of healthcare trainees across various programs and training stages, in response to the COVID-19 outbreak, is unknown.

Materials and methods

Study design

For peer review only - http://bmjopen.bmj.com/site/about/guidelines.xhtml

We conducted a cross-sectional study of healthcare trainees from the West China School of Medicine and West China Hospital, Sichuan University during February 7th-13th, 2020. We invited 7177 individuals, including 2483 undergraduates, 2606 postgraduates, and 2088 residents, to participate in this study to assess their mental health and working conditions during the COVID-19 outbreak via WeChat, a popular social media application in China. The 4184 trainees who agreed to participate were included in the analyses. For data protection, answers to these electronic questionnaires were kept anonymously. The response rates for undergraduates, postgraduates, and residents were 73.22%, 71.49%, and 24.09%, respectively (Supplementary Figure 1).

We focused exclusively on the main concerns and needs of undergraduates and postgraduates who were not involved in clinical work during the COVID-19 epidemic, and the impact of their experiences on their future career plans. We also conducted a short survey of clinical workers about work-family conflict and support during the epidemic. The total number of participants included 1818 undergraduates, 1863 postgraduates, and 503 residents. This study was approved by the Ethics Committee of Sichuan University, and electronic consent forms were obtained from all participants.

Most of the undergraduate and postgraduate students were at home throughout the country during the COVID-19 outbreak due to the Chinese Spring Festival, while all residents remained in Chengdu, Sichuan Province because of their clinical duties. As of February 6th, 2020, the total number of confirmed COVID-19 cases was 344 in Sichuan Province (102 in Chengdu), and 7226 individuals were under medical observation[10].

Healthcare programs and training stages

For peer review only - http://bmjopen.bmj.com/site/about/guidelines.xhtml

Healthcare training programs in China mainly consist of medicine, medical technology, and nursing for the preparation of future doctors, medical technologists (including medical laboratory technologists, imaging technologists, physical therapists, and optometrists.), and nurses to practice in healthcare settings. The training stages in this study were divided into three categories: undergraduate, postgraduate, and residency. All training programs begin during students' enrollment in undergraduate programs; the length of training of medical programs is 5 years, and it is 4 years for medical technology and nursing programs. After graduation, individuals continue training in a postgraduate program (3-6 years) with a primary focus on research, which can be combined with clinical training. Students who pursue careers as clinicians enter a residency program (3 years for medicine and 2 years for medical technology and nursing) for supervised clinical practice after graduation from an undergraduate or postgraduate program.

Due to the co-occurrence of COVID 19 and the Chinese Spring Festival, individuals in the early stage of training had a low proportion of clinical experiences at the time of the survey. In order to protect students without clinical experience, the medical school canceled clinical practicums for undergraduates after the COVID-19 outbreak, and a few of the senior undergraduates with internship experiences volunteered to remain at the hospital and support its clinical work. The clinically active trainees included 503 residents and 325 students (304 postgraduates and 21 undergraduates). To assess the work status of the trainees, we asked all participants in the survey, "Are you actively performing clinical duties at this time?" (Supplementary Supplemental Text).

Assessment of outbreak-related psychological distress

When we assessed psychological distress and acute stress reaction (ASR), we phrased the

For peer review only - http://bmjopen.bmj.com/site/about/guidelines.xhtml

questions so they were specific to the COVID-19 outbreak (Supplementary Supplemental Text).

Psychological distress was assessed using the Chinese version of the Kessler Psychological Distress Scale (K6). The instrument consists of six items pertaining to major depression and generalized anxiety disorder and asks respondents how frequently they have experienced relevant symptoms during the past month[11]. Each item has five options ranging from 0 (never) to 4 (all of the time), and the total score ranges from 0 to 24. We considered a score ≥5 as clinically significant distress in accordance with the validation studies on Asian populations [12, 13]. The Cronbach's alpha was 0.91 in our study, indicating good scale reliability.

The variable ASR was evaluated using the Chinese version of the Impact of Event Scale-Revised (ISE-R)[14]. The instrument consists of 22 items and yields a total score and scores on the Intrusion, Avoidance, and Hyperarousal subscales. Respondents identify a stressful event and how much they were distressed or bothered during the past seven days by the difficulties listed in the items. Responses are rated on a 5-point scale, ranging from 0 (not at all) to 4 (extremely). Individuals with a score ≥24 points are considered to have probable ASR [15, 16]. The Cronbach's α was 0.91 in our study, suggesting good scale reliability.

Assessment of the outbreak's impact

To shed light on the impact of the outbreak on trainees' lives and work, we assessed the concerns and needs of trainees without active clinical duties during the outbreak and the pandemic's influence on their future career choices. We also evaluated work-family conflict

For peer review only - http://bmjopen.bmj.com/site/about/guidelines.xhtml

and support among the trainees with clinical duties (Supplementary Supplemental Text).

Concerns and needs during the outbreak, and their influence on future career choices: To understand trainees' main concerns and needs, we asked the following question. "Under the current circumstances, I am concerned about a) being infected with the novel coronavirus; b) my physical health condition; c) my psychological health; d) academic performance; e) my social life and work; f) my traveling plans; g) the risk of infection from family members or friends; h) my personal and family's financial situation; and i) other issues." We also asked participants to respond to the following item. "If I were to work during the outbreak, I would need: a) personal protective equipment; b) social insurance; c) salary incentives; d) clinical practice guidance; e) professional track record; and f) other needs." Multiple responses were allowed for these questions. We used one single question: "Has the outbreak affected your future career plans?" to assess the impact of the COVID-19 outbreak on trainees' future career plans.

Work-family conflict and support: The 9-item Chinese version of the Work-Family Conflict and Support Scale was used to investigate work-family conflict, social support, and policy support[17]. Each dimension has three items and each item has three options: 1 (agree), 2 (neutral), and 3 (disagree).

Statistical Analysis

We compared the baseline characteristics of the trainees across the different programs (i.e., medicine, medical technology, and nursing) using Student's *t*-test (for continuous variables) and the Chi-square test (for categorical variables). We described the distributions of the symptoms' scores (transformed z-scores are reported as mean standard deviation), and the

For peer review only - http://bmjopen.bmj.com/site/about/guidelines.xhtml

proportion of identified cases (corresponding to the cut-off points stated in the Methods section), in each of the three program groups. Differences in symptom scores or the probability of cases were estimated using linear regression (β coefficients) and logistic regression (odds ratios, ORs), respectively. We examined the associations of the concerns, needs, and future career choices with psychological distress and ASR among the participants without active clinical duties, and the associations of family-work conflict with psychological distress and ASR in the participants with active clinical duties. All models were adjusted for age, sex, marital status and epidemic contact characteristics to address confounding by these variables. We also adjusted the model for training program and training stage when analyzing the associations of concerns, needs, career impact and family-work conflicts with psychological distress and ASR. As the status of clinical duty is strongly correlated with training stage, we didn't adjust for active clinical duty (yes or no) as covariates. Individuals with missing data on the measures of psychological distress (462, 11.04%) or ASR (433, 10.35%) were not included in the corresponding analyses. We analyzed the data anonymously, and all analyses were conducted using R 3.6.1; $p<0.05$ was considered to be statistically significant.

Patient and public involvement

Patients and/or the public were not involved in the design, or conduct, or reporting, or dissemination plans of this research.

Results

Demographic characteristics

The mean age of the 4184 participants was 23.41±3.81 years and 64.89% were females. No confirmed cases of COVID-19 were reported, but 31 (0.74%) trainees had at least one

For peer review only - http://bmjopen.bmj.com/site/about/guidelines.xhtml

relative who was infected. Approximately one-fifth (19.79%) of participants were involved in active clinical duties; among them, 74 (8.94%) were working on the front lines and 38 (4.59%) had direct contact with patients with COVID-19 (Table 1). Compared with trainees in the medical technology and nursing programs, the trainees in medicine were older ($p<0.01$), and more likely to be postgraduates ($p<0.01$), males ($p<0.01$), married ($p<0.01$), and living outside of Hubei Province, the first epicenter ($p=0.02$). At the time of the survey, nursing trainees were more likely to have active clinical duties and work on the front lines ($p<0.01$).

Outbreak-related psychological distress and ARS

During the outbreak, 1150 (30.90%) trainees reported significant psychological distress and 403 (10.74%) reported probable ASR. Distress symptoms (β 0.24, 95%CI 0.14 - 0.35) and cases of significant distress (OR 1.54, 95%CI 1.22 - 1.95; Table 2) were positively associated with being medical trainees, compared to being nursing trainees. A similar pattern was found among medical technology trainees (β 0.13, 95% 0.02 - 0.25; OR 1.25, 95%CI 0.97 - 1.62), although the association with distress cases was not significant. Compared with undergraduates, postgraduates (OR 1.55, 95%CI 1.16 - 2.08) in the medical program had higher levels of distress, whereas nursing residents (OR 0.38, 95%CI 0.20 - 0.71) reported a lower burden. No significant increase was found across training stages among the medical-technology trainees. Similar patterns, with weaker associations were observed for symptoms of ASR and probable distress cases across the training programs and stages within the programs, except for the lower burden of ASR symptoms reported by the medical residents compared to the undergraduates.

Associations of active clinical duties during the outbreak with distress symptoms (β 0.09,

For peer review only - http://bmjopen.bmj.com/site/about/guidelines.xhtml

95%CI 0.01 - 0.18) and cases of significant distress (OR 1.17, 95%CI 0.98 - 1.39; Table 3) were found. The association between active duties and distress was positive among the medical trainees (OR 1.85, 95%CI 1.47 - 2.33), but negative among the nursing trainees (OR 0.55, 95%CI 0.32 - 0.93). The association was slightly stronger among undergraduates (OR 4.20, 95%CI 1.61 - 11.70) than it was among postgraduates (OR 2.23, 95%CI 1.72 - 2.91). However, active clinical duty was negatively associated with ASR symptoms (β -0.10, 95%CI -0.19 - -0.02), and except for that finding, almost all associations of ASR across training programs and stages were not significant.

Outbreak's impact on vulnerable trainees

Among the trainees without active clinical duties during the outbreak, psychological distress was significantly associated with concerns about mental health (OR 2.41, 95%CI 1.90 - 3.04; Table 4) and demands for personal protective equipment (OR 1.51, 95%CI 1.07 - 2.16). They were more likely to consider future careers outside of medicine (OR 2.89, 95%CI 1.77 - 4.69). A similar pattern was found for ASR, except for concerns about academic performance, travel plans, personal or family financial hardship, and the need for personal protective equipment, which were only associated with psychological distress.

Among the trainees with active clinical duties, those with distress were more likely to report work-family conflict (ORs 2.20 - 2.68; Table 5). In contrast, adequate social support (ORs 0.42 - 0.47) and reasonable work arrangements (ORs 0.40 - 0.47) were associated with lower psychological distress. A similar pattern was found for ASR.

Discussion

In this large-scale cross-sectional study, we found that psychological distress was common

For peer review only - http://bmjopen.bmj.com/site/about/guidelines.xhtml

among healthcare trainees during the COVID-19 outbreak. Medical trainees, particularly postgraduates and those with active clinical duties, were at higher risk for psychological distress, compared to those in other training programs or at an earlier training stage. Concerns about mental health were strongly correlated with psychological distress among trainees with no clinical duties, whereas work-family conflict was the greatest concern of distressed trainees with active clinical duties.

The strain of COVID-19 on healthcare systems, medical trainees, and other practitioners is challenging. Consistent with prior research[6, 18, 19], our results indicated that psychological distress was common among healthcare trainees, especially those in medical training programs. Conversely, several studies reported that nurses providing care for confirmed or suspected COVID-19 cases had a greater mental burden than doctors[6, 20]. Given the small proportion of participants (4.59%) who had direct contact with infected patients, these inconsistent estimates do not invalidate each other.

Another important finding was the association of being in the advanced training stage and having active clinical duties with higher levels of psychological distress. Academic pressures, workload, and financial burden increase with level of training, which could, consequently, increase the mental vulnerability of senior trainees, and ultimately, contribute to negative mental outcomes[21-25]. During the COVID-19 outbreak, senior medical students and residents were encouraged to assist hospital staff with clinical work to deal with the severe workforce shortage[26, 27]. The overwhelming workload and high risk of exposure to COVID-19 might have added to their mental burden. We consistently observed a higher risk of psychological distress across all training stages among individuals who were involved in active clinical duties during the outbreak.

For peer review only - http://bmjopen.bmj.com/site/about/guidelines.xhtml

ASR often develops following direct exposure to traumatic events, such as experiencing the COVID-19 outbreak in the epicenter and being charged with the direct care (diagnosis and treatment) of patients confirmed with COVID-19[6, 28]. However, few participants were quarantined in the epicenter or had direct contact with infected patients at the time of the survey. The risk for ASR was relatively low and differences between the training programs and stages were small. Interestingly, we found that trainees with active clinical duties had fewer ASR symptoms during the outbreak.

Our findings suggest that being infected by COVID-19 was the healthcare trainees' leading concern, followed by concerns about their mental health, with regards to psychological distress and ASR. Adequate personal protective equipment and salary incentives might help reduce psychological distress, which is consistent with the finding that family-income stability is a protective factor against anxiety among medical students[29]. Among clinical workers, work-family conflict was positively associated with psychological distress, and negatively associated with social support. Therefore, adequate personal protection, timely psychological interventions, a stable financial situation, a strong family, and social support may be key factors in reducing the risk of psychological distress among healthcare trainees. Competent leadership, including active participation in outbreak preparedness and making reasonable work arrangements, could also alleviate the emotional strain on healthcare trainees, suggesting the importance of work-polices for healthcare trainees.

Studies have found that experiencing psychological distress during the training stage leads to changes in career paths[30-32]. These results are consistent with our finding that healthcare trainees who decided to work in non-medical fields in the future tended to have higher levels

For peer review only - http://bmjopen.bmj.com/site/about/guidelines.xhtml

of psychological distress, compared to the trainees determined to continue on their original paths. During the COVID-19 outbreak, healthcare trainees might have been emotionally vulnerable to the crisis, because of being knowledgeable about medicine, which increased their awareness of the dangers during the outbreak's early stages. The epidemic represents an extreme situation in which being a "doctor" is considered a demanding job with social responsibilities, which might have scared or inspired medical trainees, especially those without clinical experience. It is therefore, possible that this crisis also influenced their career choices.

Limitations

Our study has several limitations. First, given the nature of cross-sectional analyses, our data do not indicate changes in psychological distress from the pre-pandemic period; rather, they characterize the burden during the COVID-19 outbreak. Second, the response rate was low among the residents, and those who did not participate might have been the trainees with highest stress levels at work. The participation rates of undergraduate and postgraduate trainees were satisfactory (73.22% and 71.49% respectively). Such selection is not likely to provide a thorough explanation of our findings. Third, we only measured distress symptoms once in the early phase of the outbreak. Longitudinal studies are needed in the future, as symptoms may change over time. Fourth, although the effects of age, sex, training program, and training stage were adjusted for their corresponding data analyses, residual confounding remains because data on other confounding factors were inapplicable (i.e., marital status, current location, job position) or not collected (i.e. socioeconomic status). Last, although the trainees came from all parts of China, our study was conducted at a single medical school and teaching hospital. The generalizability of our findings to other hospitals and medical populations remains unclear, and therefore, needs further investigation.

For peer review only - http://bmjopen.bmj.com/site/about/guidelines.xhtml

Conclusions

Our findings suggest that psychological distress in response to the COVID-19 outbreak is common among healthcare trainees in China. Medical trainees, particularly postgraduates and with active clinical duties, were at higher risk for psychological distress than the other groups of trainees. Stress management should be provided for high-risk healthcare trainees during the outbreak, particularly if or when the training is accelerated, and trainees join the front lines of the workforce.

Acknowledgments: The authors thank Lie Zhang (West China School of Medicine of Sichuan University, Chengdu, China) for coordinating the data collection; Fenfen Ge (West China Hospital of Sichuan University, Chengdu, China), Ting Liu (West China Hospital of Sichuan University, Chengdu, China) and Xiao Liao (Southwest University, Chongqing, China) for data collection. Mr Zhang, Miss Ge, Miss Liu and Miss Liao have no conflicts of interest to declare. We also thank the participating students at the West China School of Medicine.

Contributors: Drs Zhang, Lu and Song had full access to all of the data in the study and take responsibility for the integrity of the data and the accuracy of the data analysis. Wang and Li contributed equally to the work.

Concept and design: Wang, Li, Zhang.

Acquisition, analysis, or interpretation of data: Wang, Li, Song, Feng.

Drafting of the manuscript: Wang, Li, Song, Lu.

Critical revision of the manuscript for important intellectual content: all authors.

Statistical analysis: Wang, Jiang.

For peer review only

For peer review only - http://bmjopen.bmj.com/site/about/guidelines.xhtml

Obtained funding: Zhang, Song, Lu.

Administrative, technical, or material support: Zhang, Song, Lu, Feng.

Supervision: Zhang, Song, Lu.

All authors have contributed significantly to this work and have met the qualification of authorship.

Funding: This research was supported by the National Natural Science Foundation of China (No. 81971262 to Dr. Song, No. 81801359 to Dr. Lu), Swedish Research Council (No. 2018-00648 to Dr. Lu), West China Hospital COVID-19 Epidemic Science and Technology Project (No. HX-2019-nCoV-014 to Dr. Song, No. HX-2019-nCoV-019 to Dr. Zhang), and Sichuan University Emergency Grant (No. 2020scunCoVyingji1002 to Dr. Song, No. 2020scunCoVyingji1005 to Dr. Zhang).

Competing interests: All authors declare no competing interests.

Patient and public involvement: Patients and/or the public were not involved in the design, or conduct, or reporting, or dissemination plans of this research.

Ethics approval: This study was approved by the Ethics Committee of the Sichuan University.

Data availability statement: The data that support the findings of this study are available from the corresponding author, WZ, upon reasonable request.

Reference

For peer review only - http://bmjopen.bmj.com/site/about/guidelines.xhtml

1. WHO. World Health Organization: Coronavirus disease (COVID-19) outbreak situation. World Health Organization, 2020.
2. Shi L, Lu Z-A, Que J-Y, Huang X-L, Liu L, Ran M-S, et al. Prevalence of and Risk Factors Associated With Mental Health Symptoms Among the General Population in China During the Coronavirus Disease 2019 Pandemic. JAMA network open. 2020; 3(7): e2014053-e.
3. Shechter A, Diaz F, Moise N, Anstey DE, Ye S, Agarwal S, et al. Psychological distress, coping behaviors, and preferences for support among New York healthcare workers during the COVID-19 pandemic. General hospital psychiatry. 2020; 66: 1-8.
4. Temsah M-H, Al-Sohime F, Alamro N, Al-Eyadhy A, Al-Hasan K, Jamal A, et al. The psychological impact of COVID-19 pandemic on health care workers in a MERS-CoV endemic country. J Infect Public Health. 2020; 13(6): 877-82.
5. Zerbini G, Ebigbo A, Reicherts P, Kunz M, Messman H. Psychosocial burden of healthcare professionals in times of COVID-19 - a survey conducted at the University Hospital Augsburg. Ger Med Sci. 2020; 18: Doc05-Doc.
6. Lai J, Ma S, Wang Y, Cai Z, Hu J, Wei N, et al. Factors Associated With Mental Health Outcomes Among Health Care Workers Exposed to Coronavirus Disease 2019. JAMA network open. 2020; 3(3): e203976-e.
7. Chew NWS, Lee GKH, Tan BYQ, Jing M, Goh Y, Ngiam NJH, et al. A multinational, multicentre study on the psychological outcomes and associated physical symptoms amongst healthcare workers during COVID-19 outbreak. Brain, behavior, and immunity. 2020; 88: 559-65.
8. Baker DM, Bhatia S, Brown S, Cambridge W, Kamarajah SK, A K, et al. Medical student involvement in the COVID-19 response. Lancet. 2020; 395(10232): 1254.
9. Bauchner H, Sharfstein J. A Bold Response to the COVID-19 Pandemic: Medical Students, National Service, and Public Health. JAMA. 2020.
10. Health Commission of Sichuan Province C. The latest situation of new coronavirus pneumonia in Sichun province (released on February 7, 2020). (ed HCoS Province). Available from: https://dwz.cn/Y1p9dhTl, 2020.
11. Kang Y-k, Guo W-J, Xu H, Chen Y-h, Li X-j, Tan Z-p, et al. The 6-item Kessler psychological distress scale to survey serious mental illness among Chinese undergraduates: Psychometric properties and prevalence estimate. Comprehensive Psychiatry. 2015; 63.
12. Furukawa TA, Kawakami N, Saitoh M, Ono Y, Nakane Y, Nakamura Y, et al. The performance of the Japanese version of the K6 and K10 in the World Mental Health Survey Japan. International Journal of Methods in Psychiatric Research. 2008; 17(3): 152-8.
13. Kessler RC, Green JG, Gruber MJ, Sampson NA, Bromet E, Cuitan M, et al. Screening for serious mental illness in the general population with the K6 screening scale: results from the WHO World Mental Health (WMH) survey initiative (vol 19, pg 4, 2010). International Journal of Methods in Psychiatric Research. 2011; 20(1): 62-.
14. Wu KK, Chan KS. The development of the Chinese version of Impact of Event Scale--Revised (CIES-R). (0933-7954 (Print)).
15. Lai YH, Lee YH, Chen YR, Hung YJ. Psychometric Testing of the Impact of Event Scale-Revised

For peer review only - http://bmjopen.bmj.com/site/about/guidelines.xhtml

(IES-R) Chinese Version and Current Status in Three Major Types of Cancer Survivors in Taiwan. Psycho-Oncology. 2014; 23: 156-.

16. Imsiragic AS, Begic D, Martic-Biocina S. Acute Stress and Depression 3 Days after Vaginal Delivery - Observational, Comparative Study. Collegium Antropologicum. 2009; 33(2): 521-7.

17. HUANG D, YIN W, ZHANG Y, WANG P, SUN K. Effects of Social and Policy Supports on Work-family Conflict in Physicians. Chinese Mental Health Journal. 2009; 23(01): 27-32.

18. Liu C, Yang Y-z, Zhang XM, Xu X, Dou Q-L, Zhang W-W. The prevalence and influencing factors for anxiety in medical workers fighting COVID-19 in China: A cross-sectional survey. medRxiv. 2020: 2020.03.05.20032003.

19. Zhang W-R, Wang K, Yin L, Zhao W-F, Xue Q, Peng M, et al. Mental Health and Psychosocial Problems of Medical Health Workers during the COVID-19 Epidemic in China. Psychotherapy and psychosomatics. 2020: 1-9.

20. Liu ZR, Han B, Jiang RM, Huang YQ, Ma C, Wen J, et al. Mental Health Status of Doctors and Nurses During COVID-19 Epidemic in China (3/4/2020). Available from: http://dxdoiorg/102139/ssrn3551329.

21. Dyrbye L, Thomas M, Shanafelt T. Medical Student Distress: Causes, Consequences, and Proposed Solutions. Mayo Clinic proceedings Mayo Clinic. 2006; 80: 1613-22.

22. Chunming WM, Harrison R, MacIntyre R, Travaglia J, Balasooriya C. Burnout in medical students: a systematic review of experiences in Chinese medical schools. BMC Medical Education. 2017; 17(1): 217.

23. Levey RE. Sources of stress for residents and recommendations for programs to assist them. Acad Med. 2001; 76(2): 142-50.

24. Dyrbye LN, Thomas MR, Massie FS, Power DV, Eacker A, Harper W, et al. Burnout and suicidal ideation among U.S. medical students. Ann Intern Med. 2008; 149(5): 334-41.

25. Moffat KJ, McConnachie A Fau - Ross S, Ross S Fau - Morrison JM, Morrison JM. First year medical student stress and coping in a problem-based learning medical curriculum. (0308-0110 (Print)).

26. Stokes DC. Senior Medical Students in the COVID-19 Response: An Opportunity to Be Proactive. Academic emergency medicine : official journal of the Society for Academic Emergency Medicine. 2020; 27(4): 343-5.

27. Services CfMM. Teaching Hospitals, Teaching Physicians and Medical Residents: CMS Flexibilities to Fight COVID-19. (ed CfMM Services): 2020.

28. Sun L, Sun Z, Wu L, Zhu Z, Zhang F, Shang Z, et al. Prevalence and Risk Factors of Acute Posttraumatic Stress Symptoms during the COVID-19 Outbreak in Wuhan, China. medRxiv. 2020: 2020.03.06.20032425.

29. Cao W, Fang Z, Hou G, Han M, Xu X, Dong J, et al. The psychological impact of the COVID-19 epidemic on college students in China. Psychiatry research. 2020; 287: 112934.

30. Melincavage SM. Student nurses' experiences of anxiety in the clinical setting. (1532-2793 (Electronic)).

31. Hawkins M. 2017 Final Year Medical Residents Survey. 2018.

32. Scharer S, Freitag A. Physicians' exodus: why medical graduates leave Austria or do not work in

For peer review only - http://bmjopen.bmj.com/site/about/guidelines.xhtml

clinical practice. (1613-7671 (Electronic)).

For peer review only

For peer review only - http://bmjopen.bmj.com/site/about/guidelines.xhtml

Table 1 Characteristics of healthcare trainees – N (%) or mean (SD).

	All	Medicine	Medical technology	Nursing	P
**Participants, N**	4184	2727 (65.18)	944 (22.56)	513 (12.26)	
**Age, years, mean (SD)**	23.41 (3.81)	24.20 (3.84)	21.90 (3.24)	21.98 (3.36)	<0.01
**Sex**					<0.01
Male	1469 (35.11)	1133 (41.55)	265 (28.07)	71 (13.84)	
Female	2715 (64.89)	1594 (58.45)	679 (71.93)	442 (86.16)	
**Marital status**					<0.01
Married	331 (7.91)	254 (9.31)	48 (5.08)	29 (5.65)	
Unmarried	3853 (92.09)	2473 (90.69)	896 (94.92)	484 (94.35)	
**Training stage**					<0.01
Undergraduate	1791 (42.81)	940 (34.47)	588 (62.29)	263 (51.27)	
Postgraduate	1890 (45.17)	1662 (60.95)	142 (15.04)	86 (16.76)	
Residency	503 (12.02)	125 (4.58)	214 (22.67)	164 (31.97)	
**Location**					0.02
Hubei [a]	67 (1.60)	46 (1.69)	20 (2.12)	1 (0.19)	
Outside Hubei	4117 (98.40)	2681 (98.31)	924 (97.88)	512 (99.81)	
**Relatives with COVID-19**					0.90
No	4153 (99.26)	2706 (99.23)	937 (99.26)	510 (99.42)	
Yes	31 (0.74)	21 (0.77)	7 (0.74)	3 (0.58)	
**Active clinical duty**					<0.01
No	3356 (80.21)	2301 (84.38)	719 (76.17)	336 (65.50)	
Yes	828 (19.79)	426 (15.62)	225 (23.83)	177 (34.50)	
**Working position [b]**					<0.01
Frontline [c]	74 (8.94)	36 (8.54)	5 (2.22)	33 (18.64)	
Second-line	754 (91.06)	390 (91.55)	220 (97.78)	144 (81.36)	
**Contact with COVID-19 [b]**					0.11
Yes	38 (4.59)	24 (5.63)	11 (4.89)	3 (1.69)	
No	790 (95.41)	402 (94.37)	214 (95.11)	174 (98.31)	

a. Hubei Province was the epicenter at the time of the survey.
b. Information was only assessed for participants with activity clinical duty.
c. Frontline working positions was defined as working in departments directly engaging in care for patients with COVID-19, including Emergency, Respiratory, Critical Care Medicine, and Infectious Disease Departments.

For peer review only

For peer review only - http://bmjopen.bmj.com/site/about/guidelines.xhtml

Table 2 Psychological distress and Acute stress reaction among healthcare trainees between different training programs and stages.

	Psychological distress [a]				Acute stress reaction [b]			
	Symptoms (z-score)		Cases		Symptoms (z-score)		Cases (403)	
	Mean±SD	β (95%CI) [c]	N (%)	OR (95%CI) [c]	Mean±SD	β (95%CI) [c]	N (%)	OR (95%CI) [c]
Training program								
Medicine	0.06±1.05	0.24 (0.14 - 0.35)	792 (33.22)	1.54 (1.22 - 1.95)	0.01±1.01	0.14 (0.04 - 0.24)	256 (10.66)	1.08 (0.78 - 1.52)
Medical technology	-0.07±0.92	0.13 (0.02 - 0.25)	242 (27.94)	1.25 (0.97 - 1.62)	0.03±0.99	0.13 (0.02 - 0.24)	96 (11.00)	1.06 (0.74 - 1.54)
Nursing	-0.18±0.85	Ref.	116 (24.58)	Ref.	-0.08±0.95	Ref.	51 (10.71)	Ref.
Medicine								
By training stage								
Undergraduate	-0.10±0.99	Ref.	223 (26.05)	Ref.	-0.01±1.06	Ref.	86 (9.92)	Ref.
Postgraduate	0.16±1.08	0.23 (0.09 - 0.37)	529 (37.23)	1.55 (1.16 - 2.08)	0.02±0.99	0.05 (-0.08 - 0.19)	163 (11.39)	1.14 (0.74 - 1.78)
Residency	0.10±0.90	0.14 (-0.09 - 0.37)	40 (37.38)	1.45 (0.90 - 2.31)	-0.07±0.84	-0.08 (-0.31 - 0.15)	7 (6.73)	0.58 (0.22 - 1.31)
Medical technology								
By training stage								
Undergraduate	-0.07±0.97	Ref.	155 (28.23)	Ref.	0.06±1.05	Ref.	66 (11.98)	Ref.
Postgraduate	-0.02±0.82	-0.14 (-0.40 - 0.12)	36 (30.00)	0.83 (0.44 - 1.55)	0.01±0.90	-0.09 (-0.37 - 0.19)	11 (8.80)	0.62 (0.23 - 1.56)
Residency	-0.10±0.84	-0.15 (-0.35 - 0.04)	51 (25.89)	0.73 (0.45 - 1.17)	-0.06±0.86	-0.15 (-0.36 - 0.06)	19 (9.64)	0.72 (0.36 - 1.43)
Nursing								
By training stage								
Undergraduate	-0.10±0.90	Ref.	75 (29.88)	Ref.	0.05±1.05	Ref.	32 (12.75)	Ref.
Postgraduate	-0.02±0.96	0.06 (-0.29 - 0.41)	21 (29.58)	0.92 (0.35 - 2.30)	0.04±0.88	-0.04 (-0.42 - 0.33)	8 (10.67)	0.77 (0.18 - 2.81)
Residency	-0.39±0.67	-0.25 (-0.46 - -0.05)	20 (13.33)	0.38 (0.20 - 0.71)	-0.36±0.73	-0.39 (-0.62 - -0.16)	11 (7.33)	0.55 (0.23 - 1.28)

a. In this analysis, 462 (11.04%) individuals who missed the measure of psychological distress were not included.
b. In this analysis, 433 (10.35%) individuals who missed the measure of acute stress reaction were not included.
c. Estimates were adjusted for age, sex (male or female), marital status (married or unmarried), location (Hubei or outside Hubei), and relatives with COVID-19 (yes or no).

For peer review only - http://bmjopen.bmj.com/site/about/guidelines.xhtml

Table 3 Psychological distress and Acute stress reaction among healthcare trainees with and without active clinical duty.

	Psychological distress [a]				Acute stress reaction [b]			
	Symptoms (z-score)		Cases		Symptoms (z-score)		Cases	
	Mean±SD	β (95%CI) [c]	N (%)	OR (95%CI) [c]	Mean±SD	β (95%CI) [c]	N (%)	OR (95%CI) [c]
Active clinical duty								
No	-0.03±0.99	Ref.	882 (29.73)	Ref.	0.02±1.02	Ref.	326 (10.84)	Ref.
Yes	0.12±1.02	0.09 (0.01 - 0.18)	268 (35.50)	1.17 (0.98 - 1.39)	-0.07±0.92	-0.10 (-0.19 - -0.02)	77 (10.36)	0.93 (0.71 - 1.22)
By training program								
Medicine								
No duty	-0.00[d]±1.02	Ref.	609 (30.53)	Ref.	0.01±1.02	Ref.	212 (10.47)	Ref.
With duty	0.39±1.10	0.36 (0.24 - 0.47)	183 (47.04)	1.85 (1.47 - 2.33)	-0.00[d]±0.98	-0.01 (-0.12 - 0.11)	44 (11.67)	1.12 (0.77 - 1.58)
Medical technology								
No duty	-0.08±0.94	Ref.	184 (27.84)	Ref.	0.04±1.02	Ref.	76 (11.38)	Ref.
With duty	-0.06±0.86	-0.04 (-0.20 - 0.12)	58 (28.29)	0.94 (0.64 - 1.38)	-0.03±0.88	-0.08 (-0.25 - 0.09)	20 (9.76)	0.89 (0.50 - 1.57)
Nursing								
No duty	-0.10±0.90	Ref.	89 (28.62)	Ref.	0.03±1.01	Ref.	38 (12.06)	Ref.
With duty	-0.33±0.75	-0.19 (-0.37 - -0.01)	27 (16.77)	0.55 (0.32 - 0.93)	-0.30±0.78	-0.30 (-0.49 - -0.10)	13 (8.07)	0.69 (0.32 - 1.40)
By training stage								
Undergraduate								
No duty	-0.10±0.96	Ref.	442 (26.98)	Ref.	0.02±1.05	Ref.	181 (10.96)	Ref.
With duty	0.54±1.18	0.62 (0.17 - 1.08)	11 (61.11)	4.20 (1.61 - 11.70)	-0.05±1.16	0.01 (-0.49 - 0.51)	3 (16.67)	1.75 (0.39 - 5.58)
Postgraduate								
No duty	0.06±1.02	Ref.	440 (33.11)	Ref.	0.01±0.97	Ref.	145 (10.69)	Ref.
With duty	0.52±1.14	0.49 (0.35 - 0.62)	146 (51.59)	2.23 (1.72 - 2.91)	0.06±1.03	0.07 (-0.06 - 0.20)	37 (13.50)	1.35 (0.90 - 1.97)
Residency								
No duty	-	Ref.	-	Ref.	-	Ref.	-	Ref.
With duty	-0.15±0.82	-	111 (24.45)	-	-0.16±0.82	-	37 (8.20)	-

a. In this analysis, 462 (11.04%) individuals who missed the measure of psychological distress were not included.
b. In this analysis, 433 (10.35%) individuals who missed the measure of acute stress reaction were not included.
c. Estimates were adjusted for age, sex (male or female), marital status (married or unmarried), location (Hubei or outside Hubei), and relatives with COVID-19 (yes or no).
d. -0.00: < -0.01

For peer review only - http://bmjopen.bmj.com/site/about/guidelines.xhtml

Table 4. Associations of concerns and needs during COVID-19 outbreaks with psychological distress and Acute stress reaction among healthcare trainees without active clinical duty.

	Psychological distress [a]			Acute stress reaction [b]		
	No (n=2085)	Yes (n=882)		No (n=2682)	Yes (n=326)	
	N (%)	N (%)	OR (95%CI) [c]	N (%)	N (%)	OR (95%CI) [c]
**Concerns**						
Being infected with the novel coronavirus	1298 (66.84)	644 (33.16)	1.33 (1.09 - 1.61)	1713 (87.00)	256 (13.00)	1.42 (1.05 - 1.95)
Physical health condition	458 (59.17)	316 (40.83)	1.41 (1.16- 1.72)	630 (80.36)	154 (19.64)	1.81 (1.37 - 2.39)
Psychological health	189 (46.78)	215 (53.22)	2.41 (1.90 - 3.04)	294 (71.01)	120 (28.99)	3.24 (2.42- 4.31)
Academic performance	1195 (66.24)	609 (33.76)	1.30 (1.09 - 1.56)	1606 (87.95)	220 (12.05)	0.97 (0.74 - 1.27)
Social life and work	383 (64.37)	212 (35.63)	1.19 (0.95 - 1.48)	505 (83.61)	99 (16.39)	1.38 (1.01 - 1.87)
Traveling plans	141 (58.02)	102 (41.98)	1.36 (1.01 - 1.82)	201 (81.38)	46 (18.62)	1.22 0.81 - 1.79)
Family members or friends being infected with the novel coronavirus	1297 (68.41)	599 (31.59)	0.94 (0.78 - 1.13)	1688 (87.96)	231 (12.04)	0.91 (0.69 - 1.21)
Personal and family's financial situation	249 (59.29)	171 (40.71)	1.27 (1.01 - 1.61)	353 (82.48)	75 (17.52)	1.18 (0.85 - 1.61)
**Needs**						
Personal protective equipment	1907 (69.50)	837 (30.50)	1.51 (1.07 - 2.16)	2485 (89.26)	299 (10.74)	0.74 (0.48 - 1.18)
Social insurance	1819 (69.24)	808 (30.76)	1.31 (0.98 - 1.76)	2371 (89.03)	292 (10.97)	0.86 (0.58 - 1.32)
Salary incentives	1386 (68.21)	646 (31.79)	1.37 (1.12 - 1.67)	1809 (87.69)	254 (12.31)	1.79 (1.32 - 2.44)
Clinical practice guidance	1317 (70.58)	549 (29.42)	0.89 (0.73 - 1.08)	1672 (88.42)	219 (11.58)	1.13 (0.85 - 1.50)
Professional track record	1213 (70.16)	516 (29.84)	0.91 (0.75 - 1.11)	1551 (88.63)	199 (11.37)	0.92 (0.69 - 1.23)
**Future career choice**						
Healthcare worker	1256 (73.80)	446 (26.20)	Ref.	1587 (91.89)	140 (8.11)	Ref.
Medicine-related - but not bedside	618 (69.52)	271 (30.84)	1.26 (1.05 - 1.52)	791 (87.89)	109 (12.11)	1.54 (1.17 - 2.02)
Outside of medicine	36 (50.70)	35 (49.30)	2.89 (1.77 - 4.69)	51 (72.86)	19 (27.14)	4.10 (2.29 - 7.09)
Indeterminate	175 (57.38)	130 (42.62)	2.11 (1.64 - 2.72)	253 (81.35)	58 (18.65)	2.57 (1.82 - 3.58)

a. In this analysis, 389 (11.59%) individuals who missed the measure of psychological distress were not included.
b. In this analysis, 348 (10.37%) individuals who missed the measure of acute stress reaction were not included.
c. Estimates were adjusted for age, sex (male or female), marital status (married or unmarried), location (Hubei or outside Hubei), relatives with COVID-19 (yes or no), training programs (medicine, medical technology or nursing), and training stage (undergraduate, postgraduate or residency).

For peer review only - http://bmjopen.bmj.com/site/about/guidelines.xhtml

Table 5 Associations of family-work conflicts during COVID-19 outbreak with psychological distress and Acute stress reaction among healthcare trainees with active clinical duty.

	Psychological distress [a]			Acute stress reaction [b]		
	No (n=487)	Yes (n=268)		No (n=666)	Yes (n=77)	
	N (%)	N (%)	OR (95%CI) [c]	N (%)	N (%)	OR (95%CI) [c]
**Work-family conflict**						
**Difficult to care for family due to work**						
Agree	122 (18.32)	28 (36.36)	2.53 (1.67 - 3.84)	59 (12.11)	93 (34.70)	1.86 (1.06 - 3.22)
Neutral	331 (49.70)	42 (54.55)	Ref.	249 (5.13)	135 (50.37)	Ref.
Disagree	213 (31.98)	7 (9.09)	0.47 (0.31 - 0.72)	179 (36.76)	40 (14.93)	0.25 (0.10 - 0.55)
**Family responsibilities affected work**						
Agree	37 (5.56)	14 (18.18)	2.20 (1.16 - 4.29)	19 (3.90)	33 (12.31)	2.56 (1.22 – 5.20)
Neutral	265 (39.79)	44 (57.14)	Ref.	172 (35.32)	145 (54.10)	Ref.
Disagree	364 (54.65)	19 (24.88)	0.39 (0.28 - 0.56)	296 (60.78)	90 (33.58)	0.31 (0.17 - 0.54)
**Difficulties in juggling work and family**						
Agree	36 (5.41)	14 (18.18)	2.68 (1.35 - 5.56)	14 (2.87)	37 (13.81)	2.44 (1.13 - 5.12)
Neutral	246 (36.94)	43 (55.84)	Ref.	155 (31.83)	140 (52.24)	Ref.
Disagree	384 (57.66)	20 (25.97)	0.37 (0.26 - 0.53)	318 (65.30)	91 (33.96)	0.28 (0.15 - 0.50)
**Social support**						
**Support from family**						
Agree	466 (69.97)	35 (45.45)	0.47 (0.33 - 0.67)	359 (73.72)	148 (55.22)	0.41 (0.24 - 0.71)
Neutral	166 (24.92)	31 (40.26)	Ref.	107 (21.97)	95 (35.45)	Ref.
Disagree	34 (5.11)	11 (14.29)	1.16 (0.59 - 2.33)	21 (4.31)	25 (9.33)	1.62 (0.70 - 3.55)
**Support from colleagues**						
Agree	545 (81.83)	44 (57.14)	0.42 (0.28 - 0.63)	414 (85.01)	181 (67.54)	0.32 (0.19 - 0.55)
Neutral	110 (16.52)	30 (38.96)	Ref.	65 (13.35)	81 (30.22)	Ref.
Disagree	11 (1.65)	3 (3.90)	0.51 (0.15 - 1.70)	8 (1.64)	6 (2.24)	0.99 (0.21 - 3.57)
**Support from supervisors**						
Agree	505 (75.83)	30 (38.96)	0.42 (0.29 - 0.60)	389 (79.98)	150 (55.97)	0.22 (0.13 - 0.38)
Neutral	146 (21.92)	39 (50.65)	Ref.	87 (17.86)	105 (39.18)	Ref.
Disagree	15 (2.25)	8 (10.39)	0.86 (0.35 - 2.14)	11 (2.26)	13 (4.85)	2.32 (0.85 – 6.04)
**Policy support**						

For peer review only - http://bmjopen.bmj.com/site/about/guidelines.xhtml

	Psychological distress [a]			Acute stress reaction [b]		
	No (n=487)	Yes (n=268)		No (n=666)	Yes (n=77)	
**Reasonable holiday arrangement**						
Agree	463 (69.52)	28 (36.36)	0.44 (0.30 - 0.65)	369 (75.77)	126 (47.01)	0.27 (0.15 - 0.48)
Neutral	156 (23.42)	34 (44.16)	Ref.	94 (19.30)	102 (38.06)	Ref.
Disagree	47 (7.06)	15 (19.48)	1.32 (0.72 - 2.44)	24 (4.93)	40 (14.93)	1.51 (0.72 – 3.07)
**Reasonable duty arrangement**						
Agree	468 (70.27)	29 (37.66)	0.47 (0.32 - 0.68)	371 (76.18)	129 (48.13)	0.27 (0.15 - 0.47)
Neutral	163 (24.47)	37 (48.05)	Ref.	100 (20.53)	106 (39.55)	Ref.
Disagree	35 (5.26)	11 (14.29)	1.69 (0.86 - 3.40)	16 (3.29)	33 (12.31)	1.27 (0.56 - 2.73)
**Flexible policies to balance family and work**						
Agree	438 (65.77)	25 (32.47)	0.40 (0.28 - 0.58)	356 (73.10)	109 (40.67)	0.29 (0.16 - 0.52)
Neutral	200 (30.03)	36 (46.75)	Ref.	120 (24.64)	123 (45.90)	Ref.
Disagree	28 (4.20)	16 (20.78)	3.09 (1.52 - 6.74)	11 (2.26)	36 (13.43)	3.47 (1.63 – 7.24)

a. In this analysis, 85 (10.27%) individuals who missed the measure of psychological distress were not included.

b. In this analysis, 73 (8.82%) individuals who missed the measure of acute stress reaction were not included.

c. Estimates were adjusted for age, sex (male or female), marital status (married or unmarried), relatives with COVID-19 (yes or no), working position (frontline or second-line), contact with COVID-19 (yes or no), training programs (medicine, medical technology or nursing), and training stage (undergraduate, postgraduate or residency).

For peer review only

For peer review only - http://bmjopen.bmj.com/site/about/guidelines.xhtml

Supplementary

Figure 1 Research Flow Chart

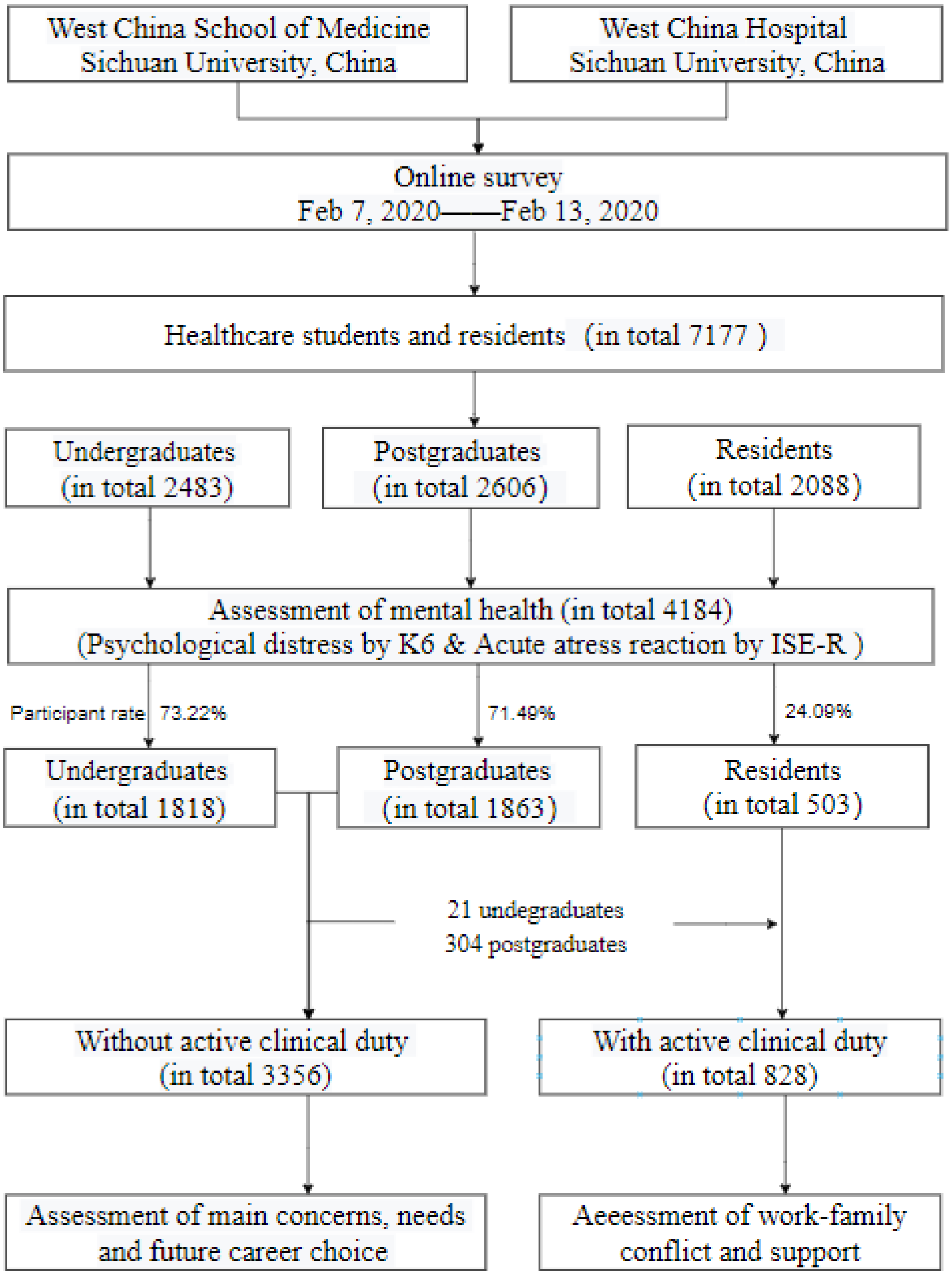

Supplemental Text:

Demographic information

a) Age: ________ years
b) Sex: A male, B female
c) Training stage: A undergraduate, B postgraduate, C residency
d) Training program: A medicine, B medical technology, C nursing
e) Marital status: A married, B unmarried
f) Location: _______ (current)
g) Have you had relatives infected with COVID-19?
A. No B. Yes
h) Are you actively performing clinical duties at this time?
A. No B. Yes

<u>If Yes, go on:</u>

i) Working position: _________ department.
ii) Since the outbreak, have you contacted with COVID-19 patients?
A. Yes B. No
iii) Work-family conflict and support
Since the outbreak,

a) my current job has made it difficult for me to care for my family.
b) family responsibilities have affected my work.
c) I had difficulties in juggling work and family.
d) I can get support from my family.
e) I can get support from my colleagues.
f) I can get support from my leader.
g) The hospital's holiday arrangement is reasonable.
h) The hospital's duty arrangement is reasonable.
i) The hospital has had a flexible policy that allowed me to juggle family and work.

<u>If No, go on:</u>

i) Concerns during the outbreak (Multiple choices)
Under the current circumstances, I am concerned about

a) being infected with the novel coronavirus;
b) my physical health condition;
c) my psychological health;
d) academic performance;
e) my social life and work;
f) my traveling plans;
g) the risk of infection from family members or friends;
h) my personal and family's financial situation;
i) other issues.

ii) Needs during the outbreak (Multiple choices)

For peer review only - http://bmjopen.bmj.com/site/about/guidelines.xhtml

If I were to work during the outbreak, I would need

a) personal protective equipment;
b) social insurance;
c) salary incentives;
d) clinical practice guidance;
e) professional track record;
f) other needs.

iii) The influence on future career choice (single question)

Has the outbreak affected your future career plans? i) Healthcare worker; ii) Medicine-related, but not bedside; iii) Outside of medicine; iv) Indeterminate.

Psychological distress

Since the COVID-19 outbreak,

a) How often did you feel nervous?
b) How often did you feel hopeless?
c) How often did you feel restless or fidgety?
d) How often did you feel so depressed that nothing could cheer you up?
e) How often did you feel that everything was an effort?
f) How often did you feel worthless?

Acute stress reaction

During the past seven days concerning the COVID-19 outbreak, how much have you been distressed or bothered by these difficulties?

a) Any reminder brought back feelings about it.
b) I had trouble staying asleep.
c) Other things kept making me think about it.
d) I felt irritable and angry.
e) I avoided letting myself get upset when I thought about it or reminded me of it.
f) I thought about it when I did not mean to.
g) I felt as if it had not happened or was not real.
h) I stayed away from reminders of it.
i) Pictures about it popped into my mind.
j) I was jumpy and easily startled.
k) I tried not to think about it.
l) I was aware that I still had many feelings about it, but I did not deal with them.
m) My feelings about it were kind of numb.
n) I found myself acting or feeling like I was back at that time.
o) I had trouble falling asleep.
p) I had waves of strong feelings about it.
q) I tried to remove it from my memory.
r) I had trouble concentrating.
s) Reminders of it caused me to have physical reactions, such as sweating, trouble breathing, nausea, or a pounding heart.

For peer review only

t) I had dreams about it.
u) I felt watchful and on-guard.
v) I tried not to talk about it.

For peer review only

For peer review only - http://bmjopen.bmj.com/site/about/guidelines.xhtml

STROBE Statement—Checklist of items that should be included in reports of ***cross-sectional studies***

	Item No	Recommendation	Page No
**Title and abstract**	1	(*a*) Indicate the study's design with a commonly used term in the title or the abstract	1
		(*b*) Provide in the abstract an informative and balanced summary of what was done and what was found	3-4
**Introduction**			
Background/rationale	2	Explain the scientific background and rationale for the investigation being reported	6
Objectives	3	State specific objectives, including any prespecified hypotheses	6
**Methods**			
Study design	4	Present key elements of study design early in the paper	6-7
Setting	5	Describe the setting, locations, and relevant dates, including periods of recruitment, exposure, follow-up, and data collection	7
Participants	6	(*a*) Give the eligibility criteria, and the sources and methods of selection of participants	7-8
Variables	7	Clearly define all outcomes, exposures, predictors, potential confounders, and effect modifiers. Give diagnostic criteria, if applicable	8-10
Data sources/ measurement	8*	For each variable of interest, give sources of data and details of methods of assessment (measurement). Describe comparability of assessment methods if there is more than one group	6-11
Bias	9	Describe any efforts to address potential sources of bias	10-11
Study size	10	Explain how the study size was arrived at	7
Quantitative variables	11	Explain how quantitative variables were handled in the analyses. If applicable, describe which groupings were chosen and why	10-11
Statistical methods	12	(*a*) Describe all statistical methods, including those used to control for confounding	10-11
		(*b*) Describe any methods used to examine subgroups and interactions	Not applicable
		(*c*) Explain how missing data were addressed	11; 23-27 (Table2-5)
		(*d*) If applicable, describe analytical methods taking account of sampling strategy	Not applicable
		(*e*) Describe any sensitivity analyses	Not applicable
**Results**			
Participants	13*	(a) Report numbers of individuals at each stage of study—eg numbers potentially eligible, examined for eligibility, confirmed eligible, included in the study, completing follow-up, and analysed	7, 11-12; 22 (Table 1); Supplementary
		(b) Give reasons for non-participation at each stage	7
		(c) Consider use of a flow diagram	Supplementary
Descriptive data	14*	(a) Give characteristics of study participants (eg demographic, clinical, social) and information on exposures and potential confounders	11-12; 22 (Table 1);

For peer review only - http://bmjopen.bmj.com/site/about/guidelines.xhtml

		(b) Indicate number of participants with missing data for each variable of interest	11; 23-27 (Table 2-5)
Outcome data	15*	Report numbers of outcome events or summary measures	11; 23-27 (Table 1-5)
Main results	16	(*a*) Give unadjusted estimates and, if applicable, confounder-adjusted estimates and their precision (eg, 95% confidence interval). Make clear which confounders were adjusted for and why they were included	11; 23-27 (Table 2-5)
		(*b*) Report category boundaries when continuous variables were categorized	9
		(*c*) If relevant, consider translating estimates of relative risk into absolute risk for a meaningful time period	Not appliable
Other analyses	17	Report other analyses done—eg analyses of subgroups and interactions, and sensitivity analyses	Not appliable
**Discussion**			
Key results	18	Summarise key results with reference to study objectives	13-14
Limitations	19	Discuss limitations of the study, taking into account sources of potential bias or imprecision. Discuss both direction and magnitude of any potential bias	16
Interpretation	20	Give a cautious overall interpretation of results considering objectives, limitations, multiplicity of analyses, results from similar studies, and other relevant evidence	13-16
Generalisability	21	Discuss the generalisability (external validity) of the study results	16
**Other information**			
Funding	22	Give the source of funding and the role of the funders for the present study and, if applicable, for the original study on which the present article is based	18

*Give information separately for exposed and unexposed groups.

Note: An Explanation and Elaboration article discusses each checklist item and gives methodological background and published examples of transparent reporting. The STROBE checklist is best used in conjunction with this article (freely available on the Web sites of PLoS Medicine at http://www.plosmedicine.org/, Annals of Internal Medicine at http://www.annals.org/, and Epidemiology at http://www.epidem.com/). Information on the STROBE Initiative is available at www.strobe-statement.org.

For peer review only
